# Identification of *Salmonella* Pullorum Factors Affecting Immune Reaction in Macrophages from the Avian Host

**DOI:** 10.1128/spectrum.00786-23

**Published:** 2023-05-16

**Authors:** Xiao Fei, Qiuchun Li, Xinan Jiao, John Elmerdahl Olsen

**Affiliations:** a Key Laboratory of Prevention and Control of Biological Hazard Factors (Animal Origin) for Agri-Food Safety and Quality, Ministry of Agriculture of China, Yangzhou University, Yangzhou, People’s Republic of China; b Jiangsu Key Lab of Zoonosis/Jiangsu Co-Innovation Center for Prevention and Control of Important Animal Infectious Diseases and Zoonoses, Yangzhou University, Yangzhou, People’s Republic of China; c Joint International Research Laboratory of Agriculture and Agri-Product Safety, Yangzhou University, Yangzhou, People’s Republic of China; d Department of Veterinary and Animal Sciences, Faculty of Health and Medical Sciences, University of Copenhagen, Denmark; Navarrabiomed-Universidad Publica de Navarra-Complejo Hospitalario de Navarra, IdiSNA

**Keywords:** *Salmonella*, persistent infection, Th2, macrophages, Th1/Th2 responses

## Abstract

The host-specific Salmonella serovar *S*. Pullorum (SP) modulates the chicken immune response to a Th2-biased response associated with persistent infection. This is different from the Th1-biased immune response induced by the genetically close serovar, *S*. Enteritidis (SE). Based on core genome differences between SP and SE, we used three complementary bioinformatics approaches to identify SP genes, which may be important for stimulation of the immune response. Defined mutants were constructed in selected genes, and the infection potential and ability of mutants to stimulate cytokine production in avian derived HD11 macrophages were determined. Deletion of large genomic regions unique to SP did not change infection potential nor immune stimulation significantly. Mutants in genes with conserved single nucleotide polymorphisms (SNPs) between the two serovars in the region 100 bp upstream of the start codon (conserved upstream SNPs [CuSNPs]) such as *sseE*, *osmB*, *tolQ*, a putative immune antigen, and a putative persistent infection factor, exhibited differences in induction of inflammatory cytokines compared to wild-type SP, suggesting a possible role of these CuSNPs in immune regulation. Single nucleotide SP mutants correcting for the CuSNP difference were constructed in the upstream region of *sifA* and *pipA*. The SNP corrected *pipA* mutant expressed *pipA* at a higher level than the wild-type SP strain, and the mutant differentially caused upregulation of proinflammatory cytokines. It suggests that this CuSNP is important for the suppression of proinflammatory responses. In conclusion, this study has identified putative immune stimulating factors of relevance to the difference in infection dynamics between SP and SE in avian macrophages.

**IMPORTANCE** Salmonella Pullorum is host specific to avian species, where it causes life-threatening infection in young birds. It is unknown why it is host restricted and causes systemic disease, rather than gastroenteritis normally seen with Salmonella. In the present study, we identified genes and single nucleotide polymorphisms (SNPs; relative to the broad-host-range type Salmonella Enteritidis), which affected survival and immune induction in macrophages from hens suggesting a role in development of the host specific infection. Further studies of such genes may enable understanding of which genetic factors determine the development of host specific infection by *S.* Pullorum. In this study, we developed an *in silico* approach to predict candidate genes and SNPs for development of the host-specific infection and the specific induction of immunity associated with this infection. This study flow can be used in similar studies in other clades of bacteria.

## INTRODUCTION

Some host-specific Salmonella serovars induce persistent, systemic infection, and this is associated with a different host immune response compared to that induced during Salmonella gastroenteritis or acute systemic infection (1). Salmonella enterica serovar Gallinarum (*S*. Gallinarum) is a host-specific serovar, which only infects birds. It exists in two biovars of which biovar Pullorum (*S*. Pullorum) is associated with pullorum disease. After acute infection, *S*. Pullorum may persist in the host for a long period (2, 3), and a recent study has associated this to the induction of a Th2-biased immune response (4). Therefore, *S*. Pullorum infection of the avian host may be a model to understand persistent Salmonella infection (4). However, it is currently unknown which factors of the bacteria contribute to the modulation of the immune response.

Salmonella enterica serovar Enteritidis (*S*. Enteritidis), a typical broad host Salmonella serovar, is evolutionary close to *S*. Pullorum (5, 6). We have previously identified conserved core genome genes and single-nucleotide polymorphisms (SNPs) differences between the two serovars (7). In this study, we hypothesized that one or more of such unique genes and/or SNPs may contribute to the induction of the unique immune response by *S.* Pullorum.

Life science databases have undoubtedly promoted the development of science, even though their utilization still has a great room for improvement (8). To facilitate the recording of genomic features with their biological signatures, standardized annotation terminologies were established, such as Gene Ontology (GO; http://geneontology.org/), KEGG pathways (https://www.kegg.jp/kegg/), and Clusters of Orthologous Groups (COGs) (9). Faced with a variety of databases, it is important for researchers to use them appropriately and efficiently according to their own research interests. To this end, we previously developed a bioinformatics tool, Duo, which facilitates customized functional identification of bacterial proteins based on signature databases (10). In the present study, we applied this tool to identify putative candidate proteins for induction of the differential immune response by *S.* Pullorum among the genes showing conserved SNP mutations in the region 100 bp upstream of the start codon (from now on denoted CuSNPs for conserved upstream SNPs). In addition, two genomic loci, which were previously shown to be unique to *S.* Pullorum (7), were likewise investigated for a putative role in the induction of differential immune responses. The aim of the study was to determine how modification by mutation of the unique genes and genes with CuSNPs in *S.* Pullorum affects survival in avian derived macrophages, as well as the induction of immune regulating cytokines in the macrophages.

## RESULTS

### Candidate CuSNPs identified by custom-build bioinformatics workflow.

**FIG 1:**
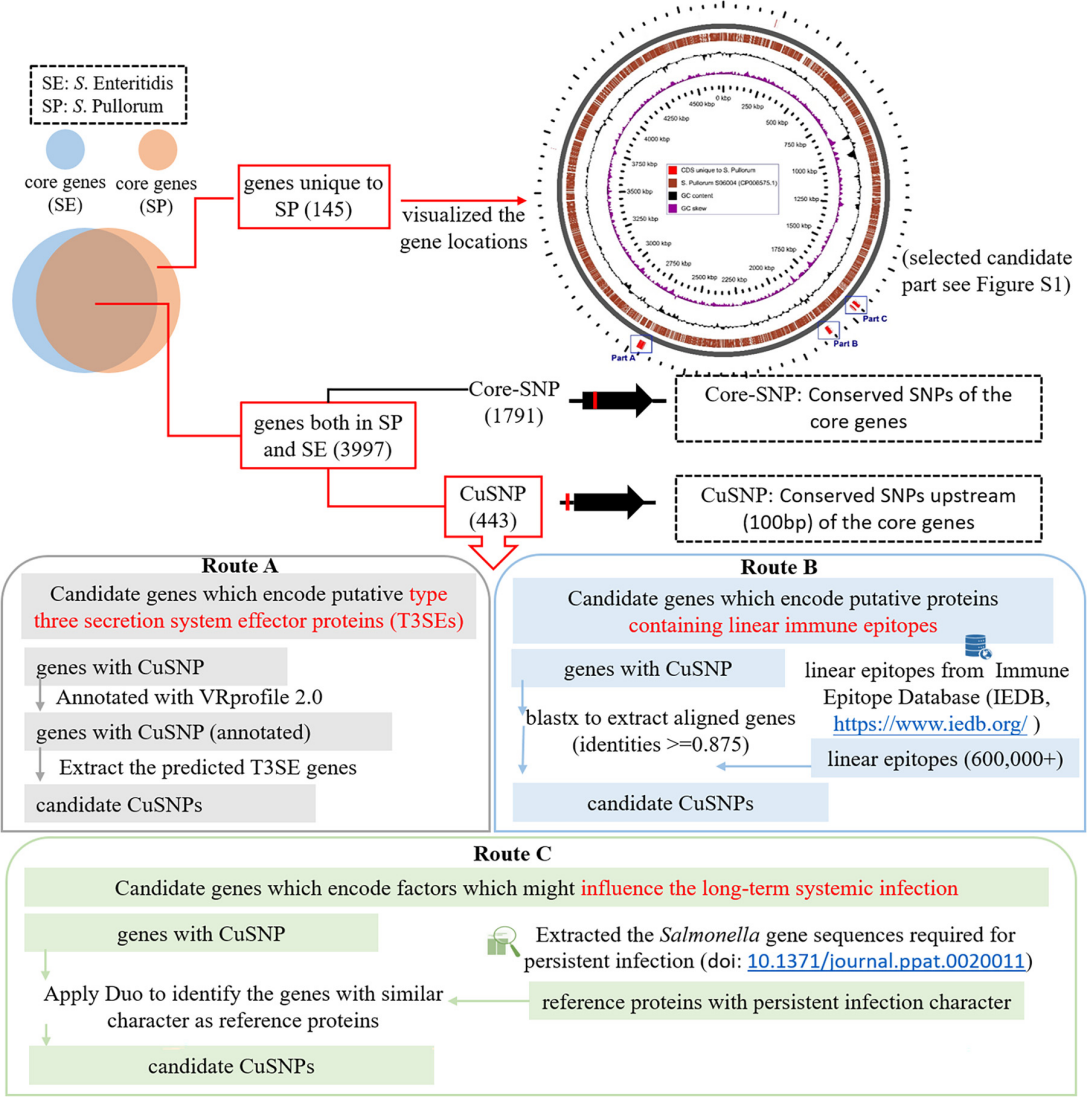
Schematic representation of *in silico* strategies to identify putative factors responsible for specific immune induction by *S*. Pullorum in the avian host.

In this study, a custom-built bioinformatics workflow was developed to identify the candidate CuSNPs that may affect immune induction by *S*. Pullorum and thus the Th2-biased immune response (Fig. 1). The workflow includes three independent routes. A total of 8 CuSNPs were identified by route A, which focused on type 3 secretion system effector proteins (T3SEs) with CuSNPs relative to *S*. Enteritidis (see Table S3), 31 were identified by route B, which focused on putative antigen epitopes in genes with CuSNPs (see Table S4), and 76 were identified by route C, which was set up to identify genes that encode factors that may modulate long-term infection in the host (see Table S5 in the supplemental material). For further analysis, a subset of these genes was extracted from these lists (Table 1).

**TABLE 1:** Genes selected for further analysis in this study among the genes identified by the custom-build bioinformatics workflow^*a*^

Tag	Preferred name (eggnog_v2)	Selection method^*b*^	Annotation (eggnog_v2)
*pipA*	*pipA*	Route A	PipA protein
*sifA*	*sifA*	Route A/route C	Sif protein
*sopB*	*sopB*	Route A	Enterobacterial virulence protein
*sptP*	*sptP*	Route A	*Yersinia* virulence determinant
*sseE*	*sseE*	Route A	Type III secretion system regulator
*glpD*	*glpD*	Route B	C-terminal domain of alpha-glycerophosphate oxidase
*group_5201*		Route B	
*group_5437*		Route B	Domain of unknown function (DUF1971)
*tolQ*	*tolQ*	Route B	MotA/TolQ/ExbB proton channel family
*group_4046*	*tia*	Route C	Opacity family porin protein
*group_6178*		Route C	SH3 domain (SH3b1 type)
*osmB*	*osmB*	Route C	Glycine zipper 2TM domain
*pagO*	*pagO*	Route C	EamA-like transporter family

a For a full list of genes identified by the bioinformatics workflow, see Tables S3 to S5 in the supplemental material.

b For route A, a bioinformatics search was set up to identify genes encoding T3SS effector molecules and which have conserved SNPs in the region 100 bp upstream of the transcription initiation site (CuSNPs) compared to the same gene in *S.* Enteritidis. For route B, a bioinformatics search was set up to identify genes in *S.* Pullorum with CuSNPs and which show homology to known immune epitopes. For route C, a bioinformatics search was set up to identify genes in *S.* Pullorum with CuSNPs and which show homology to genes known to affect persistence of *S.* Typhimurium in a mouse model of persistent infection.

### Comparison of expression of T3SE between *S*. Pullorum and *S*. Enteritidis during macrophage infection.

**FIG 2:**
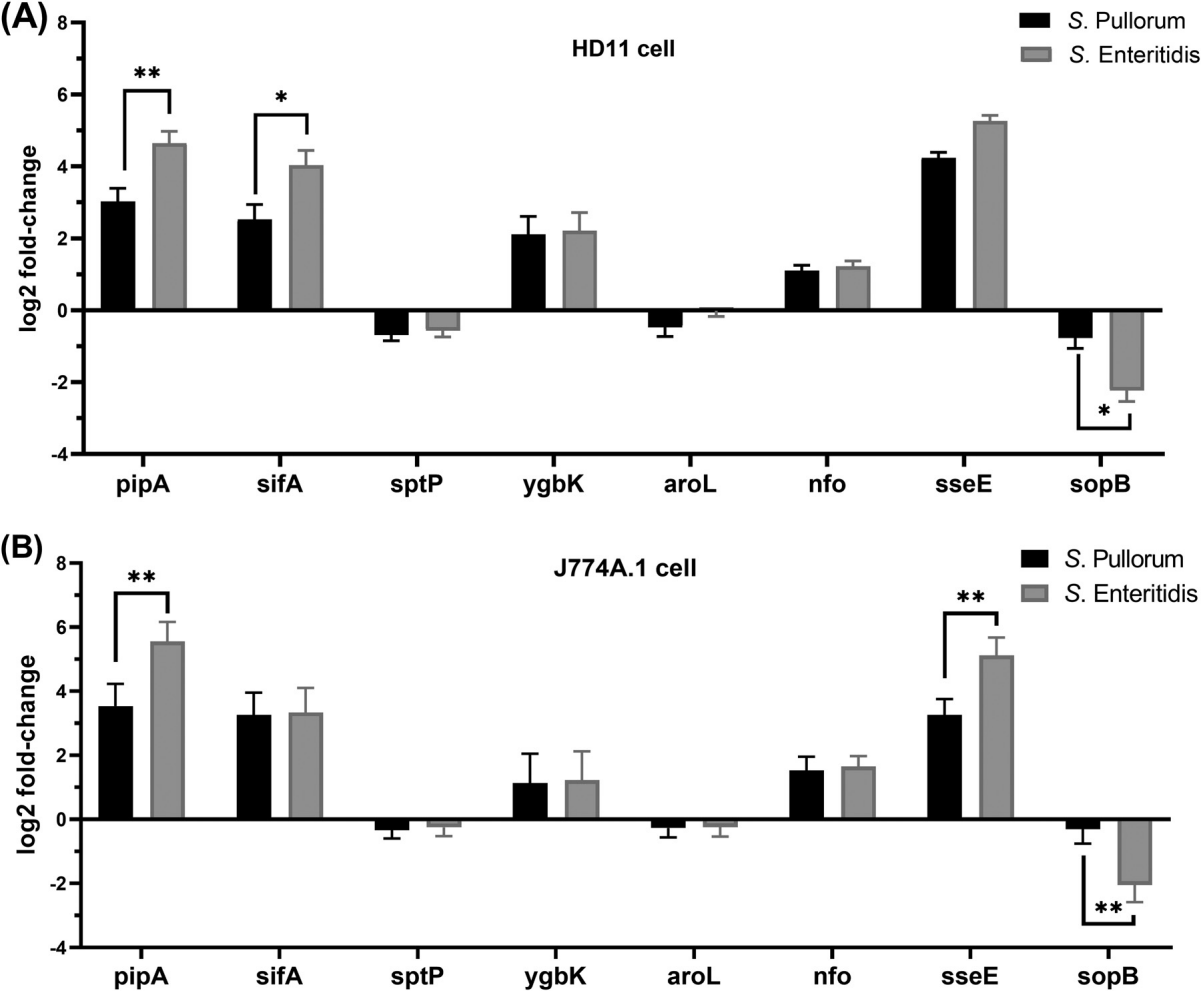
(A and B) T3SE gene expression levels (log_2_-fold change) in HD11 (A) and J774A.1 (B) cell infection (MOI of 100:1) at 6 h postinfection with *S.* Pullorum and *S.* Enteritidis compared to the expression of bacteria grown in LB media to late exponential phase (OD = 1.0). *, *P < *0.05; **, *P < *0.01.

To dissect the possible importance of the eight T3SE with CuSNPs identified in route A, we measured expression of the genes in *S*. Pullorum and *S*. Enteritidis during the infection of macrophages. The two serovars showed similar expression trends during the infection of macrophages (Fig. 2). The genes *pipA*, *sifA*, *ygbK*, *nfo*, and *sseE* were upregulated, and the genes *sptP*, *aroL*, and *sopB* were downregulated. The fold changes in expression of *pipA*, *sifA*, and *sopB*, however, were statistically different between the two serovars (Fig. 2A). The differential expression of *pipA* and *sopB* was likewise seen during J774A.1 (murine macrophages) infection, and the expression of *sseE* was further significantly different between the two serovars in this cell line (Fig. 2B).

### Uptake, survival, and cytokine responses induced in HD11 macrophages by mutants in DNA loci shown to be unique to *S.* Pullorum.

**FIG 3:**
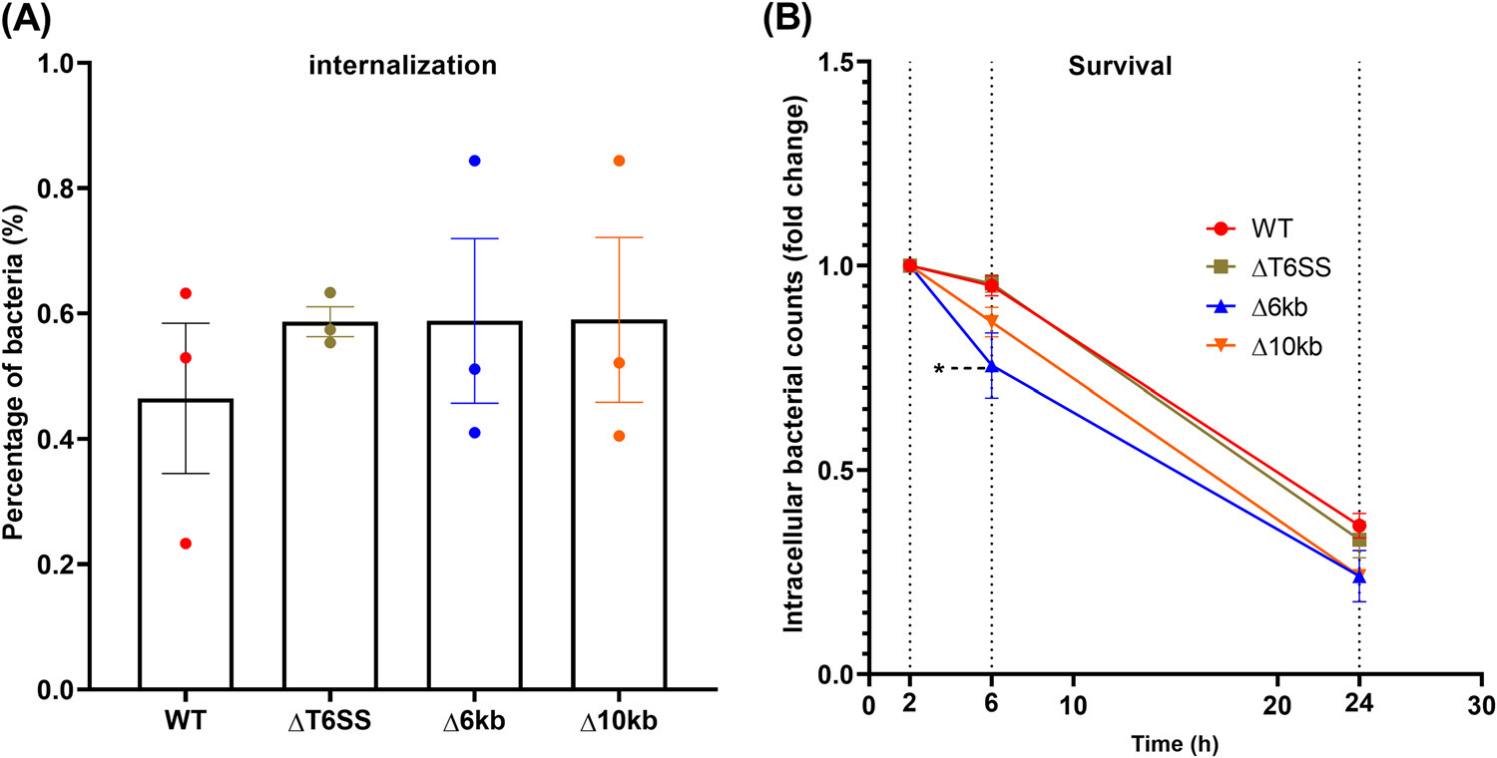
Uptake and change in intracellular counts of *S*. Pullorum mutants lacking conserved genomic parts previously shown to be absent in *S.* Enteritidis (7). (A) Percentages of cells taken up by the macrophages at 2 h postinfection. (B) Fold change in numbers of intracellular bacteria at 6 and 24 h postinfection compared to the numbers at 2 h postinfection. *, *P < *0.05.

**FIG 4:**
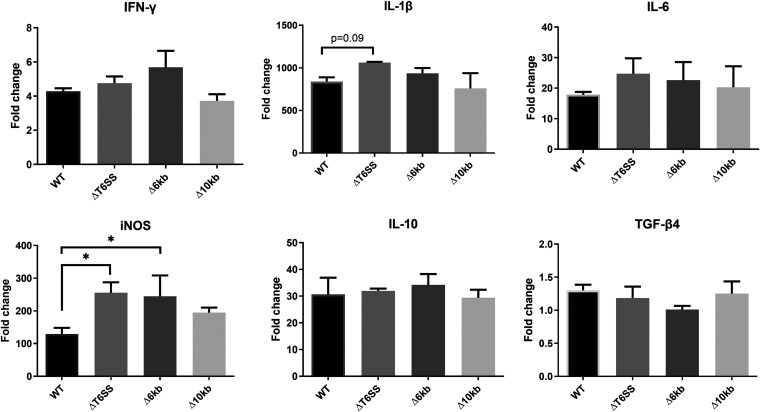
Expression of pro- and anti-inflammatory immune mediator genes in HD11 macrophages infected with *S*. Pullorum mutants lacking conserved genomic parts, which have previously been shown to be absent in *S.* Enteritidis (7) compared to expression in macrophages infected with wild-type strain at 6 h postinfection. *, *P < *0.05.

To elucidate the putative function in immune induction of two genomic loci previously shown to be present in *S*. Pullorum and absent in *S*. Enteritidis (7), we constructed three *S*. Pullorum deletion mutants (ΔT6SS, Δ6kb, and Δ10kb). As shown in Fig. 3, none of the mutants significantly changed the internalization rate in HD11 macrophages compared to the wild-type strain, just as the proliferation inside the macrophages was similar after 6 h and 24 h postinfection. The only exception was the significantly lower proliferation of the Δ6kb deletion mutant at 6 h postinfection. Both Δ6kb and Δ10kb mutants also showed lower rate of intracellular proliferation compared to the wild type (WT) at 24 h postinfection; however, this result was not significantly different from the WT strain. The slope of the curve connecting counts at 6 h postinfection to counts at 24 h postinfection showed a less-steep trend for the Δ6kb mutant, suggesting a higher ability to either grow or survive in the macrophage environment in this period. We further compared the gene expression of inflammation-related genes in the infected HD11 cells. Most of the proinflammatory cytokines (gamma interferon [IFN-γ], interleukin-1β [IL-1β], and IL-6) and anti-inflammatory cytokines (IL-10 and TGF-β4) showed no significant difference between cells infected with mutants and the WT strain (Fig. 4). The only exception was the iNOS expression in macrophages infected with the ΔT6SS and Δ6kb mutants, both of which showed significantly higher expression level than that of the WT strain.

### Role of CuSNPs in the expression of *pipA* and *sifA* in *S*. Pullorum.

**FIG 5:**
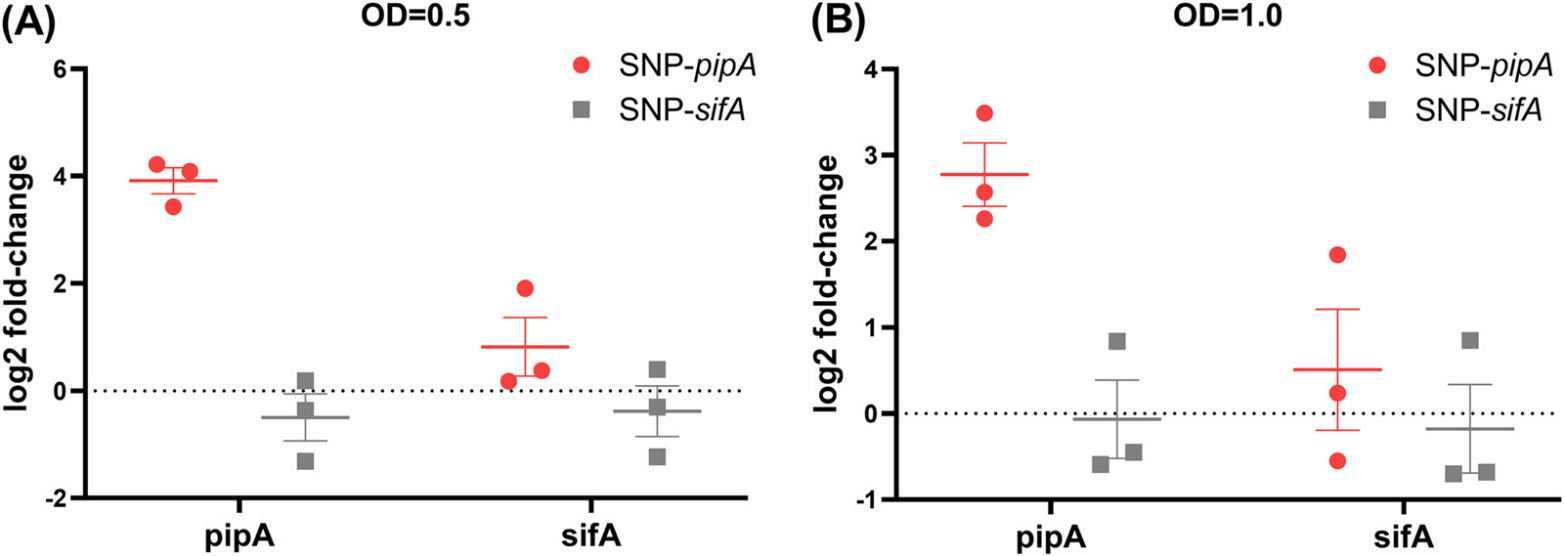
(A and B) Gene expression (log_2_-fold change) of single-nucleotide substitution mutants in *pipA* and *sifA* strains versus WT *S.* Pullorum strain measured in the midexponential phase (A) and the late exponential phase (B).

To further understand why *pipA* and *sifA* were significantly lower expressed in *S.* Pullorum compared to that in *S*. Enteritidis, we targeted the CuSNPs in these genes (SNPs shown in Fig. S2). Two single-nucleotide substitution mutants with the SNP type of *S*. Enteritidis were constructed in the *S*. Pullorum strains and are denoted SNP-*pipA* and SNP-*sifA*. The SNP-*pipA* mutant exhibited a much higher transcription level of *pipA* compared to that of the WT strain in both the middle exponential phase (Fig. 5A) and the late exponential phase (Fig. 5B), while *sifA* expression in this mutant did not differ significantly from that of the wild-type strain. The SNP-*sifA* mutant exhibited similar *pipA* and *sifA* expression as the WT strain (Fig. 5).

### Uptake and survival in HD11 macrophages of mutants in selected genes with CuSNPs and cytokine responses in macrophages infected with these mutants.

**FIG 6:**
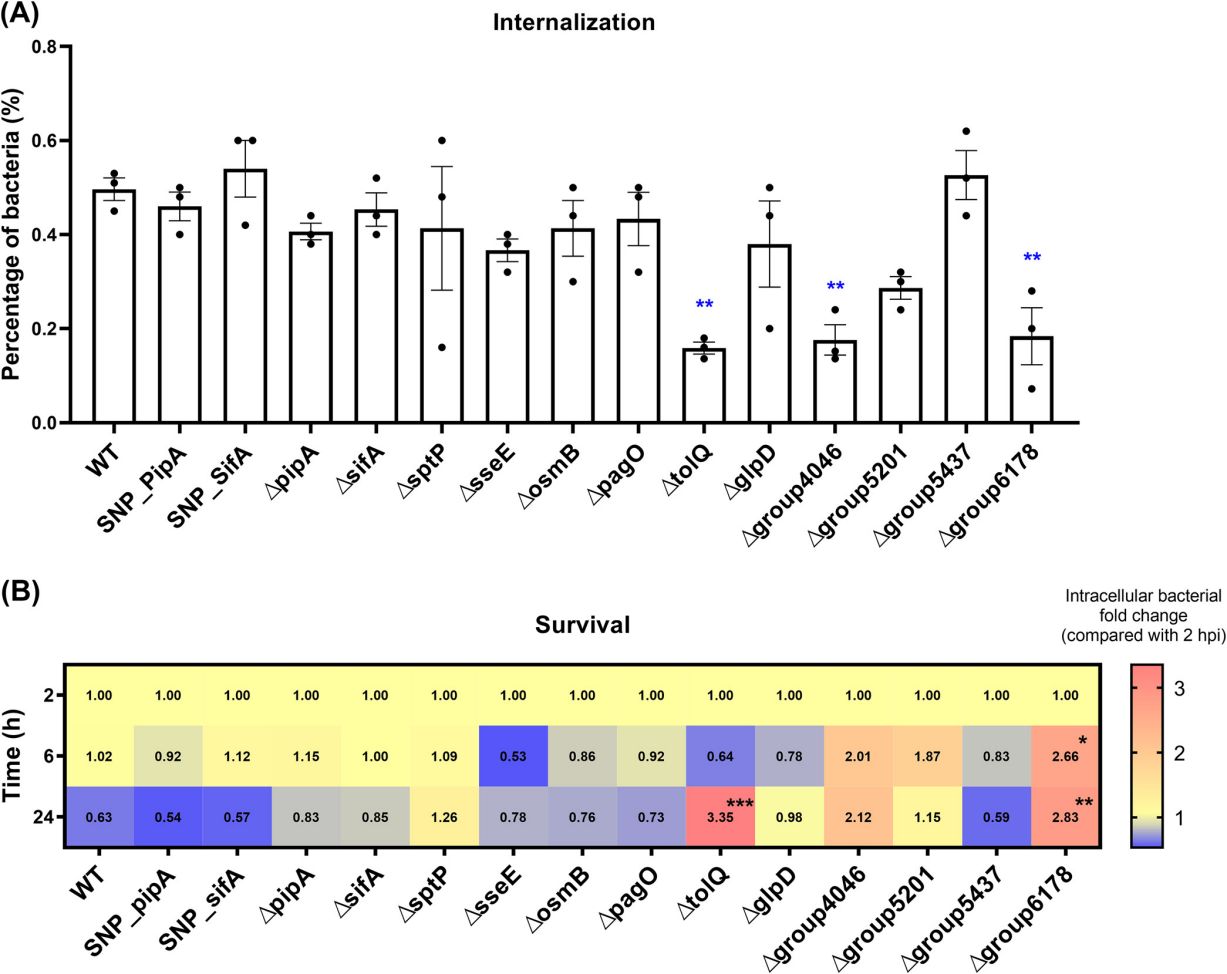
Uptake and intracellular survival of *S*. Pullorum with deletion or single nucleotide mutation in genes selected based on *in silico* analysis (Table 1). (A) Internalization percentages of the mutants at 2 h postinfection. (B) Fold change in the number of intracellular bacteria at 6 and 24 h postinfection compared to 2 h postinfection. *, *P < *0.05; **, *P < *0.01; ***, *P < *0.001.

**FIG 7:**
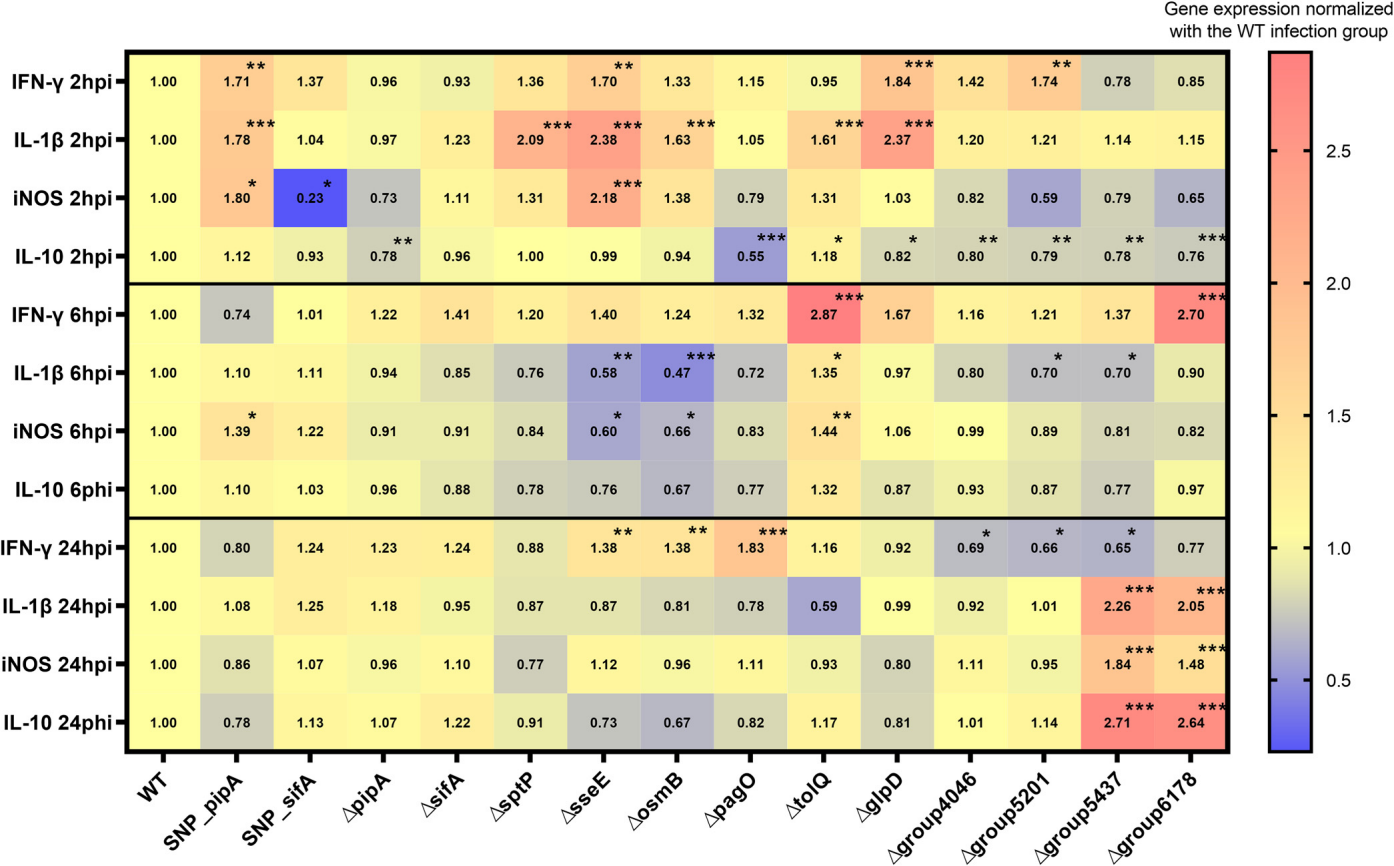
Heat map and fold changes of expression of the pro- and anti-inflammatory immune mediators in HD11 cells infected with different *S*. Pullorum mutants at 2, 6, and 24 h postinfection. The data represent the levels of gene expression normalized against the WT infection group. *, *P < *0.05; **, *P < *0.01; ***, *P < *0.001.

Twelve of the candidate genes identified in the *in silico* analysis (Table 2) were selected based on a number of criteria (see Discussion) for mutation analysis, and together with SNP-*pipA* and SNP-*sifA*, these mutants were used to infect HD11 macrophages to investigate their role in macrophage interaction and induction of cytokine responses. Three mutants (Δ*tolQ*, Δ*group4046*, and Δ*group6178*; see Table 1 for information on the two latter loci) showed significantly lower internalization rates than the WT strain (Fig. 6A), which hinted that these loci are important for *S*. Pullorum uptake in macrophages. Statistical analysis further indicated that the Δ*tolQ* and Δ*group6178* mutants showed significantly higher survival rates inside macrophages (Fig. 6B), suggesting the loci also influence the intracellular survival of *S*. Pullorum. To evaluate the cytokine responses induced by the mutants in HD11, the mRNA levels of three proinflammatory mediators (IFN-γ, IL-1β, and iNOS) and an anti-inflammatory mediator (IL-10) were measured at three time points (2, 6 and 24 h) postinfection with the mutants (Fig. 7). When expressed relatively to the WT expression at 2 h postinfection, half of the 14 mutants induced significantly higher expression of one or more of the proinflammatory mediators (IFN-γ, IL-1β, or iNOS), and half of the mutants induced a lower expression of the anti-inflammatory mediator (IL-10) (Fig. 7). SNP_*pipA* and Δ*sseE* were associated with significant upregulation of all three proinflammatory mediators, and Δ*tolQ* induced a significant upregulation of IL-10. The Δ*osmB*, Δ*sptP*, and Δ*glpD* mutants also induced upward trends for the expression of all three proinflammatory mediators, but the difference compared to the WT was not significant for all mediators. The results at 6 and 24 h postinfection clearly indicated a very dynamic regulation of expression of the selected immune modulators (Fig. 7). With a view to downregulation of proinflammatory responses and upregulation of IL-10, which would correspond to the natural *S.* Pullorum infection (4), *tolQ* was significantly associated with a reduction of the proinflammatory response after 6 h, and also the gene where mutation caused the strongest upregulation of IL-10; however, this upregulation was not significant. At 24 h postinfection, the Δ*group5437* and Δ*group6178* infection groups exhibited significantly higher levels of IL-10 (an anti-inflammatory cytokine) than the WT group; however, these two groups simultaneously showed higher levels of induction of proinflammatory mediators (IL-1β and iNOS). The reporting above was based on normalized data. As shown in Fig. S3, non-normalized gene expression levels showed that the proinflammation response was reduced from 6 h postinfection to 24 h postinfection in all the infection groups, suggesting that one should put emphasis on early regulation of this response. We further compared the survival and cytokine responses induced in HD11 macrophages by *S*. Pullorum *pipA* mutants (SNP-*pipA* and Δ*pipA*) and *S*. Enteritidis. The results are summarized in Fig. S4 and S5. *S*. Enteritidis showed a significantly higher internalization rate and concurrently a higher cytokine expression at 6 and 24 h postinfection than did the *S.* Pullorum mutants.

**TABLE 2:** *S*. Pullorum WT and mutant strains constructed in this study

Mutation type and strain	Mutation location^*a*^	Mutation target background^*b*^	
		General	Route(s)
WT strain (449/87)			
Deletion mutants lacking unique genomic clusters			
Δ6kb	2696088…2702793	Unique gene clusters in *S*. Pullorum	
Δ10kb	2709949…2720511	Unique gene clusters in *S*. Pullorum	
ΔT6SS	1852321…1867879	Unique gene clusters in *S*. Pullorum	
Single nucleotide substitution mutants			
SNP-*pipA*	1926713 (A-T mutates to C-G)	Genes with CuSNPs	Route A
SNP-*sifA*	1073868 (A-T mutates to C-G)	Genes with CuSNPs	Route A/route C
Deletion mutants lacking genes with CuSNPs			
Δ*pipA*	1926798…1927478	Genes with CuSNPs	Route A
Δ*sifA*	1072851…1073861	Genes with CuSNPs	Route A/route C
Δ*sptP*	2921499…2923106	Genes with CuSNPs	Route A
Δ*sseE*	1258880…1259296	Genes with CuSNPs	Route A
Δ*osmB*	1509396…1509614	Genes with CuSNPs	Route C
Δ*pagO*	1669433…1670347	Genes with CuSNPs	Route C
Δ*tolQ*	2195884…2196576	Genes with CuSNPs	Route B
Δ*glpD*	4154170…4155678	Genes with CuSNPs	Route B
Δ*group4046*	2678008…2678727	Genes with CuSNPs	Route C
Δ*group5201*	4495469…4496857	Genes with CuSNPs	Route B
Δ*group5437*	1619156…1619497	Genes with CuSNPs	Route B
Δ*group6178*	1791434…1792867	Genes with CuSNPs	Route C

a Mutation location indicates the mutated genomic location indexed by reference sequence CP006575.

b Mutation target background indicates the mutation target and by which screening route it was obtained. Routes A, B, and C correspond to the bioinformatics search routes described in Fig. 1.

## DISCUSSION

The host-specific serovar, *S*. Pullorum, specifically infects chickens and causes a Th2-biased immune response, as well as persistent infection (4, 11). In previous comparative studies, we have identified genomic loci which were conserved in *S*. Pullorum and did not exist in *S*. Enteritidis, as well as several conserved SNPs in coding sequences and in the regions immediately upstream of coding sequences (7). In this study, we further characterized these loci and SNPs with a view to their putative role in interaction with macrophages and the induction of immunity.

First, we selected two regions specific to *S.* Pullorum for further functional research, as they contained a high number of predicted virulence-associated genes. The results showed that the three large deletion mutants (ΔT6SS, Δ6kb, and Δ10kb) exhibited similar uptake and survival in macrophages and caused an induction of cytokine responses very similar to that of the WT strain. We concluded that most of these large conserved genomic regions were probably not essential for the differential immune responses induced by *S.* Pullorum compared to *S*. Enteritidis. We cannot rule out that genes within each of these regions regulate each other and that a single gene knockout would have yielded different results; however, we consider this unlikely. The ΔT6SS mutant disrupted most parts of the putative type 6 secretion system (T6SS) core components encoded by Salmonella pathogenicity island 19 (SPI-19) (12) but did not remove the full island. Recent research reported that deletion of the whole SPI-19 segment decreased the uptake and survival ability in macrophages (13), and their subsequent work further proved that two Sel1-like proteins encoded in SPI-19 (the surrounding loci of T6SS core components) could be the key factors causing this phenotype difference (14). Our knockout of the T6SS genes resulted in a similar phenotype, as was reported in *S*. Gallinarum for knockout of the T6SS core components (15), suggesting that macrophage phenotypes are not related to the T6SS *per se*, but rather the surrounding genes. It is currently unknown whether the *S*. Pullorum T6SS is functional. However, we did observe that mutation of the core components caused increased iNOS production from the macrophages, indicating that the T6SS downregulates this response. Further studies are needed to determine whether this is of importance for the host-specific *S.* Pullorum infection.

In addition to large horizontal DNA transmission events, SNP mutations are also important contributors to differences in pathogenicity between closely related Salmonella serovars (16, 17) because they can lead to differences in gene expression during infection (18). In the previous study, we identified a high number (*n* = 443) of CuSNPs within 100 bp upstream of conserved genes in *S*. Pullorum compared to *S.* Enteritidis (7). To select CuSNPs with a likelihood of a role in pathogenicity, the genes with CuSNPs were searched for biological signatures by three different bioinformatics analysis routes. This resulted in a list of genes with a putative role in the differential pathogenicity and immunity between the two serovars. The approach reduced the number of genes for detailed analysis considerably, and the bioinformatics workflow can be used in other bacteria for the selection of genes with a specific role in pathogenicity.

T3SEs are important factors in Salmonella host pathogen interaction, and they may contribute significantly to the induction of immune responses (19, 20). Our bioinformatics approach identified eight T3SE genes with CuSNPs. For three of these genes, *pipA*, *sifA*, and *sopB*, the CuSNP was associated with significant differences in expression of the T3SE gene between the two serovars, *S.* Pullorum and *S.* Enteritidis. Singe-nucleotide substitutions, where *S.* Pullorum sequences were manipulated to the *S.* Enteritidis sequence, were used to investigate whether the expression differences were due to the SNPs or just associated with the SNPs. Substitutions were successfully confirmed in *pipA* and *sifA*, while it was not possible to obtain the substitution in *sopB.*

The expression of *pipA* was consistently higher in the SNP-*pipA* mutant than in the WT *S*. Pullorum, suggesting that this CuSNP causes reduced *pipA* transcription. PipA family effectors—GtgA, GogA, and PipA—function as zinc metalloproteases and can dampen NF-κB signaling by cleaving p65 (21, 22). Originally, PipA was reported to be secreted by Salmonella T3SS-1 (23) and to be required for Salmonella gut pathogenicity (24). Recently, it has also been reported as a T3SS-2 effector, where it contributes to the systemic dissemination of *S.* Typhimurium in mice (25). Our study suggests that this can also be the case in *S.* Pullorum, and further studies should be carried out to clarify exactly how *pipA* is regulated and where PipA plays a role during *S*. Pullorum infection.

Cytokine expression results indicated that the SNP-*pipA* mutant induced a significantly stronger proinflammatory response than the WT strains during early macrophage infection. The total lack of Δ*pipA* expression, illustrated by a deletion mutant, resulted in immune induction similar to that of the WT strain, suggesting a threshold above which PipA upregulates proinflammatory responses. Thus, while *pipA* is dispensable for the survival of *S*. Pullorum in macrophages, the limiting effect of the SNP on *pipA* transcription might aid *S*. Pullorum in its immune evasion strategy during early stages of infection.

We selected 12 candidate genes with CuSNPs among the ones identified in the three *in silico* analyses (four T3SEs, four putative immune epitopes, and four putative persistent infection factors), and we investigated their roles in interaction with HD11 macrophages. We were interested in the genes that affected macrophage survival or where mutation caused either upregulation of proinflammatory responses or downregulation of immune-dampening IL-10. Attention first concentrated on the gene *tolQ*, which is involved in the formation of outer membrane glycerophospholipid homeostasis and which influences the survival of *S.* Typhimurium during bacteremia (26). Lacking *tolQ* in *S*. Pullorum caused different immune responses compared to the WT strain during the early stages of macrophage infection. However, since mutation of *tolQ* also caused reduced uptake and growth/survival in the macrophages, it is not possible to conclude whether this is due to the absence of this specific molecule or the absence of other molecules in the right concentration due to the lower number of intracellular bacteria. Interestingly, cell infection results also showed that *sseE* and *osmB* in *S.* Pullorum were important for limiting inflammation at 2 h postinfection, without affecting uptake and intracellular propagation. At this early infection stage, host cell receptors such as Toll-like receptors recognize pathogen-associated molecular patterns and orchestrate an efficient immune response against the pathogen (27–29). These two genes seem important to counteract the normal proinflammatory response. Then, at 6 h postinfection, the pathogen has completed cell invasion and starts to establish intracellular survival. In line with this, the two genes did not have the same dampening effect at this time point. The regulatory profile we have observed from studies of *sseE* and *osmB* mutants thus indicates that these genes contribute to the differential immunity induced by the host-specific *S*. Pullorum compared to the broad-host-range *S.* Enteritidis. How CuSNP mutations in these genes affect their expression profiles during infection should be studied further.

*S*. Pullorum exhibits persistent infection in an avian host, and this is linked to an ability to evade immunity through a long-time intracellular location (4). Therefore, in this study, we further measured the expression of immune mediators at 24 h postinfection. We hypothesize that genes with reduced inflammatory immune responses at this time could be important for the persistent infection. Mutation of two putative genes, one of which showed similar characteristics as factors involved in persistent infection of *S.* Typhimurium in mice (30) and one selected due to homology to an immune epitope in *S.* Typhi (denoted as *group6178* and *group5437* in Results), showed upregulation of proinflammatory responses. This could mean that they function to reduce immune responses. However, the results also showed that in their absence, IL-10 was upregulated, which works in the opposite direction. Further studies are needed to understand if they play a role during natural infections at late time points.

In conclusion, we identified here T3SE molecules and other factors, which are candidate genes for immune regulators assisting *S.* Pullorum in its immune evasion strategy in the avian host. We also highlight the potential significance of CuSNPs in differences between Salmonella serovars in induction of host cell cytokine responses, including a possible contribution to the Th1/Th2 differentiation during *S*. Pullorum and *S.* Enteritidis infection. The combination of bioinformatics approaches and biological analysis proved instrumental to pinpoint putative factors and could provide a model for future studies with other bacteria.

## MATERIALS AND METHODS

### *In silico* analysis to identify putative factors responsible for specific immune induction by *S*. Pullorum in avian macrophages.

In a previous study, we identified two genomic loci that were conserved in *S*. Pullorum and did not exist in *S.* Enteritidis and which contained virulence associated elements (see Fig. S1) (7). Together with 443 genes containing CuSNPs (7), these loci were further analyzed in the present study. To be able to identify putative immune regulating factors among the proteins encoded by the selected DNA, we took three different approaches (see Fig. 1). Type 3 secretion system effector proteins (T3SEs) play an important role in host-pathogen interactions (31). Therefore, as shown in route A of Fig. 1, coding sequences (CDSs) annotated as T3SEs by VRprofile web server (32) and containing CuSNPs were selected as candidate factors for further study. Immune epitopes are antigen components that the immune system recognizes. Therefore, in route B, we downloaded more than 600,000 linear epitope sequences from the Immune Epitope Database (IEDB; https://www.iedb.org/) as a comparison database and then used the BLAST 2.13.0+ program with Blastx-based method to select immune epitopes among the *S.* Pullorum CDSs that contained CuSNPs. According to previous reports, *S*. Pullorum modulates the Th2-biased immune response in the avian host, allowing for systemic and persistent infection (4, 33, 34). A random-mutation-based approach has previously been used to identify genes in the serovar *S.* Typhimurium involved in persistent infection in a mouse model (30). Therefore, in route C, we collected the sequences of these genes as a local reference database. Then CDSs with CuSNPs in *S*. Pullorum were analyzed against this collection of genes by Duo, a newly developed tool to identify functionally similar proteins (10).

### Bacterial strains and culture conditions.

*S*. Pullorum 449/87 (2) was used as the parent wild-type strain for the construction of gene-deletion and singe-nucleotide substitution mutants. *S.* Enteritidis P125109 (35) was used as the comparator strain. Bacterial strains were routinely cultured in Luria-Bertani (LB) medium or LB agar plates at 37°C, except for the strains containing temperature-sensitive plasmid pKD46, which was grown at 30°C. When appropriate, the medium was supplemented with the following antibiotics or supplements: diaminopimelic acid (DAP; 50 μg/mL), sucrose (10%), kanamycin (50 μg/mL), gentamicin (20 μg/mL), ampicillin (100 μg/mL), and chloramphenicol (Cm; 25 μg/mL).

### Mutagenesis.

Table 2 lists the mutants constructed in this study, and Table S1 lists the primers and plasmids used for mutagenesis.

### (i) Construction of deficient mutants.

Site-specific gene deletion in *S.* Pullorum 449/87 was done by inserting kanamycin cassettes using the λ Red recombination system (36). Briefly, the kanamycin cassette was obtained from pKD4, and the specific gene was replaced by the kanamycin cassette. The mutants were confirmed by PCR verification, as previously described (36).

### (ii) Construction of singe-nucleotide substitution mutants.

Single-nucleotide substitutions at the upstream of genes *pipA* and *sifA* were carried out by a scarless genome-editing technique based on the pDM4 plasmid (37, 38). Briefly, genomic DNA of *S.* Enteritidis strain P125109 was used as the PCR template to amplify DNA sequences containing the conserved SNP mutation compared to *S*. Pullorum. The fragments were confirmed by 0.8% agarose gel electrophoresis and purified by using an agarose gel DNA extraction kit (TaKaRa, Kusatsu, Shiga, Japan). Subsequently, the fragment was ligated via ClonExpress II (Vazyme, USA) into the pDM4 plasmid that was double digested with SalI and SacI. After that, the ligation products were individually transferred to DH5α strains while selecting on agar plates containing Cm (25 μg/mL). The successful recombinant plasmids (pDM4-sifA-up-SE and pDM4-pipA-up-SE) were then electroporated into the donor strain, χ7213 and selected on agar plates containing Cm (25 μg/mL) and DAP (50 μg/mL) to generate χ7213-sifA-up-SE and χ7213-pipA-up-SE. The recombinant suicide plasmids in the donor strains, χ7213-sifA-up-SE and χ7213-pipA-up-SE, were individually transferred by conjugation into *S.* Pullorum 449/87, as described previously (38). *S.* Pullorum 449/87 transconjugants which had integrated the suicide plasmid by homologous recombination were selected on LB agar plates containing 25 μg/mL Cm and were named *S.* Pullorum 449/87-sifA-up-SE and *S.* Pullorum 449/87-pipA-up-SE, respectively. To get the final single-nucleotide substitution mutants, *S.* Pullorum 449/87-sifA-up-SE and *S.* Pullorum 449/87-pipA-up-SE were subcultured at 1:100 on LB medium with 10% sucrose at 37°C overnight. Then the bacterial suspensions were spread onto LB agar plates containing 10% sucrose with appropriate dilutions and then cultured at 37°C for 24 h. Single colonies on the plates were picked up, and DNA from boiling lysates was used as the PCR templates to amplify the DNA sequences containing the SNP. To confirm the correct nucleotide mutation, the PCR product was sequenced.

### Cell infection and proliferation assay.

Cell infection and proliferation assays were performed as previously described, with minor modifications (39). Briefly, the avian macrophage cell line HD11 or the mouse-derived cell line J774 were cultured in RPMI 1640 medium (Gibco, Denmark) with 10% fetal bovine serum (FBS; Biowest, Denmark) at 37°C and 5% CO_2_. Cells were seeded with 2 × 10^5^ per well in 24-well plates 16 h prior to reaching 80% confluence for the infection assay. For the bacterial infection assay, strains were cultured to the middle exponential phase to add into cell wells at a multiplicity of infection (MOI) of 10:1, except for the infection assay for bacterial T3SE mRNA expression measurement, where an MOI of 100:1 was used to fulfill the requirement for getting enough bacterial RNA. Plates were centrifuged at 1,000 rpm for 10 min to promote interactions between bacteria and cells (time point, 0 h). After 1 h of incubation, the cells were washed twice with Dulbecco’s Phosphate Buffered Saline (DPBS) and then incubated in RPMI 1640 medium containing 100 μg/mL gentamicin and 10% FBS for another 1 h to kill the extracellular bacteria. To monitor the intracellular Salmonella proliferation, the cells were washed twice with DPBS at appropriate time points and then lysed with 0.1% Triton X-100. Then, dilutions in PBS were plated out on LB agar at 37°C for 24 h to count CFU at 2, 6, and 24 h postinfection. Three independent biological repeats of these experiments were performed.

### Quantitative real-time PCR analysis.

Cytokine mRNA expression of HD11 cells was measured at 2, 6, and 24 h postinfection, as previously reported (39). Briefly, total RNA of cells was extracted with an RNeasy minikit (Qiagen, Denmark) according to the manufacturer’s instructions. DNase (Promega, Denmark) was then used to remove the DNA contamination, and RNA was transcribed into cDNA by the GoScript reverse transcription system (Promega, Denmark) according to the manufacturer’s instructions. The cytokine mRNA expression of HD11 in the three biological infection replicates was then measured by qPCR analysis with a SYBR FastStar essential DNA Green master kit (Roche, Denmark) and a Roche LightCycler 96 real-time PCR machine with three technical replicates. The primer sequences of the cytokine genes, as well as control gene GAPDH (glyceraldehyde-3-phosphate dehydrogenase), are listed in Table S2. Measurement of the expression of T3SE genes was done using the same RNA extraction and cDNA transcription assays, and RT-qPCR analysis was carried out as previously reported (40). The primer sequences for this analysis are likewise listed in Table S2.

### Statistical analysis.

One-way or two-way analysis of variance (ANOVA) was performed with Dunnett’s multiple-comparison test to analyze significant differences in the means of expression and infection data using GraphPad Prism software (v8.3.0). A difference was considered statistically significant when the *P* value was <0.05.

### Data availability.

All data needed to evaluate the conclusions here are included in this study and the associated supplemental material. Additional data related to this study can be requested from the authors.
