# Supplementary material for: Identification of Salmonella Pullorum Factors Affecting Immune Reaction in Macrophages from the Avian Host
Source: Microbiol Spectr. 2023 May 16;11(3):e00786-23. doi: 10.1128/spectrum.00786-23 (PMC10269470; doi:10.1128/spectrum.00786-23)
Supplement: Supplemental file 1 — Supplemental material. Download spectrum.00786-23-s0001.pdf, PDF file, 1.2 MB [file spectrum.00786-23-s0001.pdf]

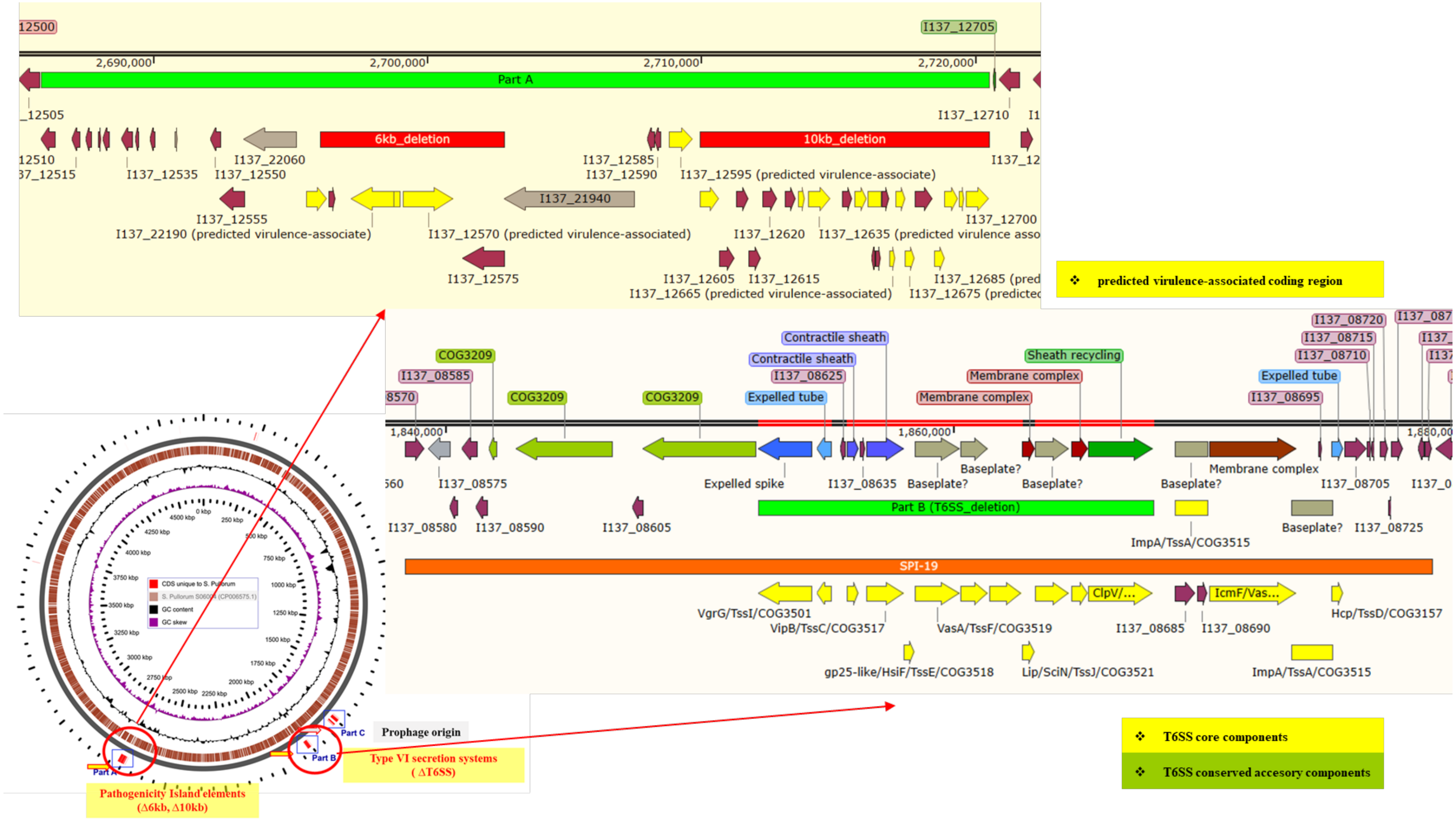

Figure S1. BLAST atlases of coding sequences (CDS) which are unique to *S*. Pullorum compared to *S*. Enteritidis. The red circles indicate the loci selected for mutation analysis in the current study.

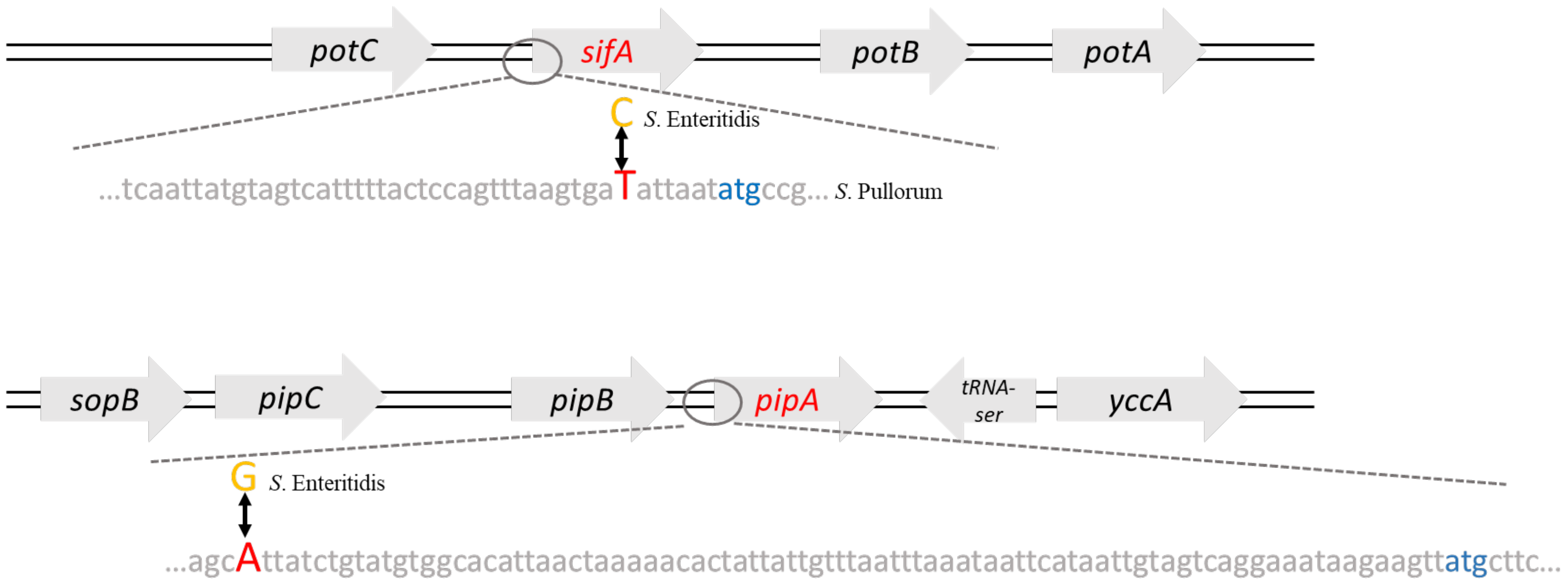

Figure S2. Conserved SNPs in the upstream region from *pipA* and *sifA* genes in *S*. Pullorum compared to *S*. Enteritidis.

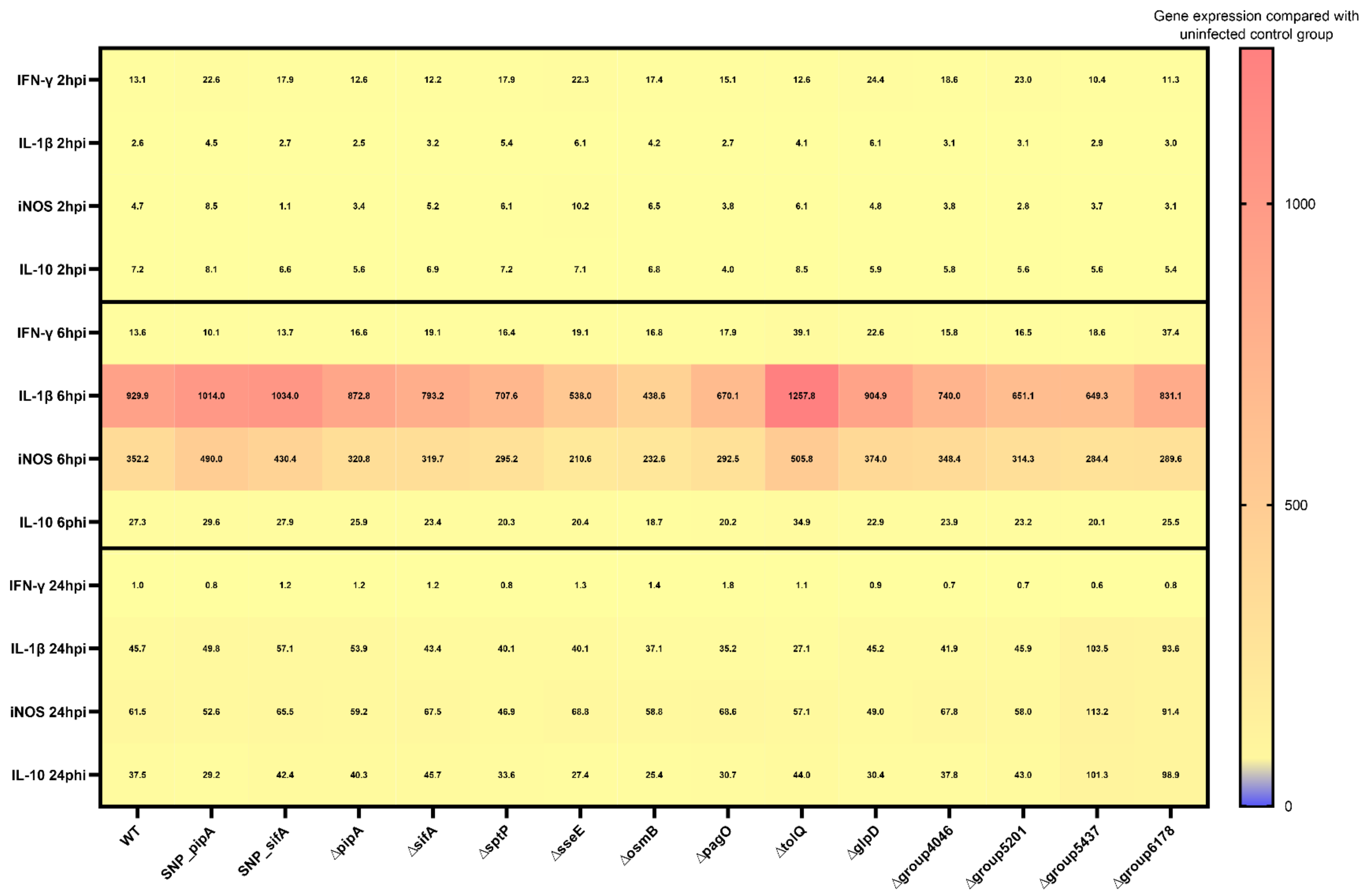

Figure S3. Heat map of the expression of pro- and anti-inflammatory immune mediators in HD11 macrophage cell at 2 h, 6 h and 24 h post-infection with *S*. Pullorum WT and mutants. Data represent levels of gene expression compared with uninfected HD11 control group.

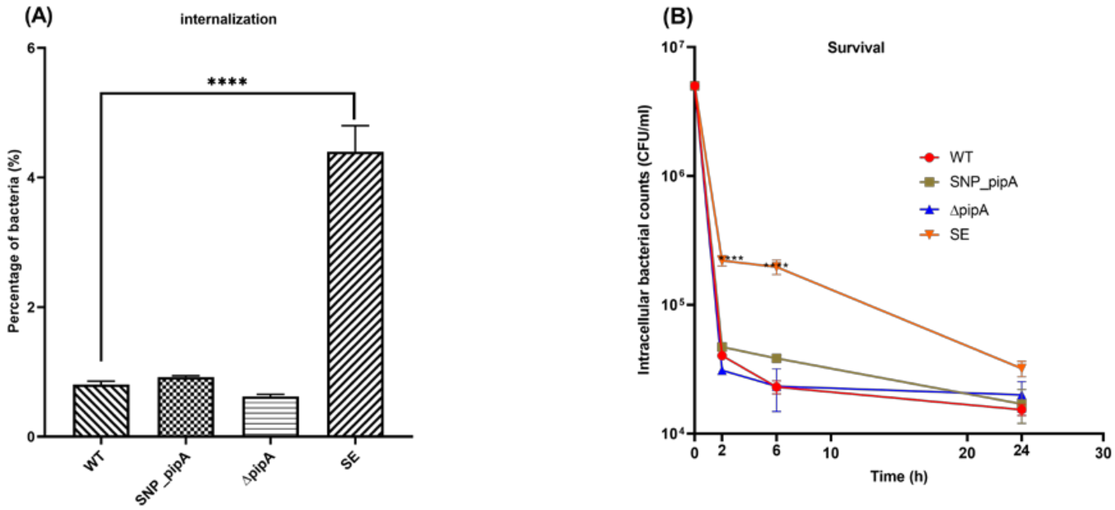

Figure S4. Uptake and change in intracellular counts of *S*. Pullorum (WT), *pipA* related mutants (SNP_*pipA* and Δ*pipA*) and *S*. Enteritidis (SE). (A) shows the percentage of cells taken up by the macrophages 2 h post-infection and (B) indicates the numbers of intracellular bacterial at 2h, 6 h and 24 h post-infection. **** $p < 0.0001$.

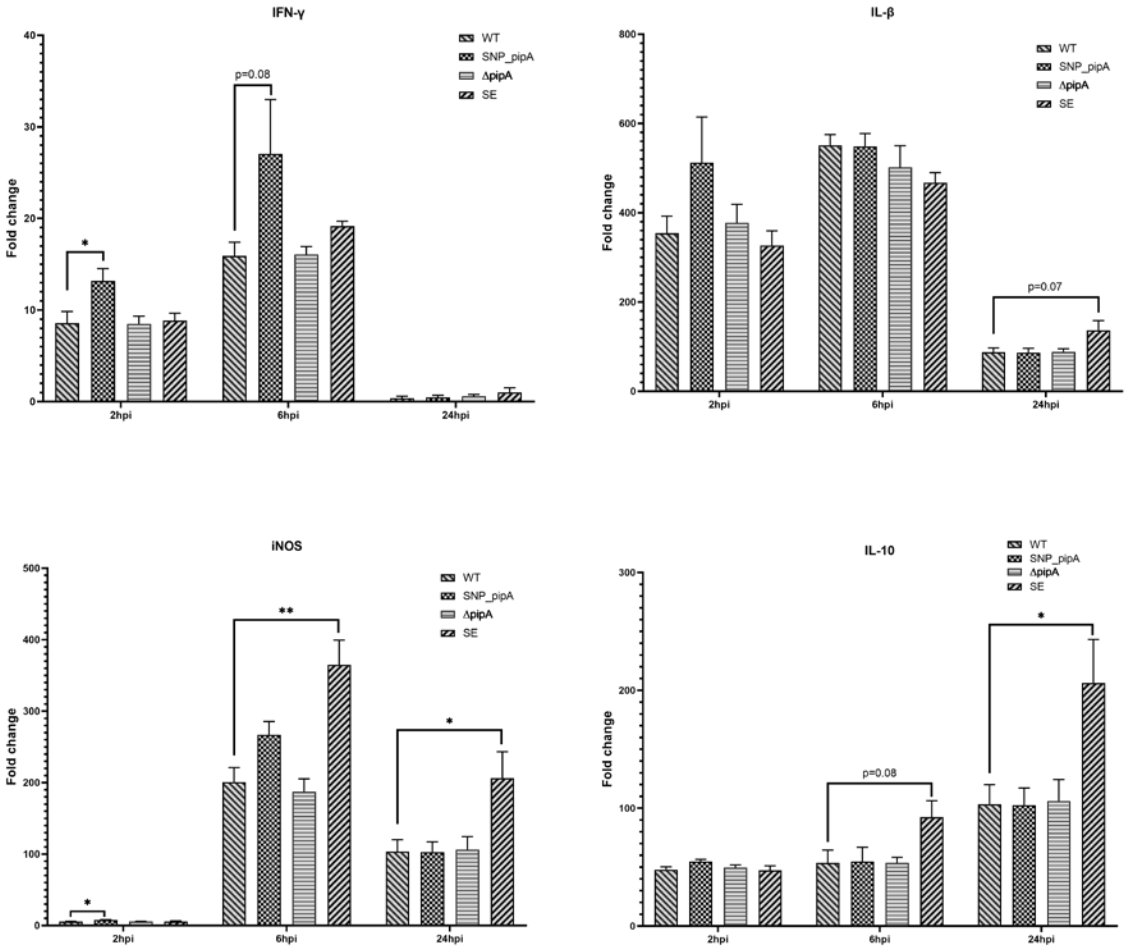

Figure S5. Expression of pro- and anti-inflammatory immune mediator genes in HD11 macrophages infected with *S*. Pullorum (WT), *pipA* related mutants (SNP_*pipA* and Δ*pipA*) and *S*. Enteritidis (SE) at 2h, 6 h and 24 h post-infection. * $p < 0.05$, ** $p < 0.01$.

Table S1. Plasmids, strains and primers for mutagenesis used in this study.

**Plasmids, strains or primers**	**Description* or sequence (5'→3')**	**Source or comments**
**Plasmids**		
pKD46	rep_{pSC101}^{ts} Gem^R $P_{araBAD\gamma\beta}$ exo	(Doublet *et al.*, 2008)
pKD4	rep_{R6K} γAmp^R FRT Kam^R FRT	(Datsenko & Wanner, 2000)
pDM4	Suicide plasmid; Cm^R	(Milton *et al.*, 1992, Yin *et al.*, 2018)
pDM4-sifA-up-SE	DNA sequence containing upstream sequence part of sifA in *S*. Enteritidis cloned into pDM4; Cm^R	This study
pDM4-pipA-up-SE	DNA sequence containing upstream sequence part of pipA in *S*. Enteritidis cloned into pDM4; Cm^R	This study
**Strains**		
χ7213 (*E. coli*)	Donor strain; Thi-1 thr-l leuB6 fhuA21 lacYl glnV44 ΔasdA4 recA1 RP4 2-Tc::Mu[Xpir]; Kam^R	(Kang *et al.*, 2002, Yin *et al.*, 2018)
DH5α (*E. coli*)	F-λ^- endA1 hsdR17 $hsdM^+$ supE44 thi-1 recA1 gyrA96 relA1Δ(argF lacZYA)U169 φ80dΔ (lacZ)M15	Life Technologies
χ7213-sifA-up-SE (*E. coli*)	χ7213 donor strain with pDM4-sifA-up-SE plasmid	This study
χ7213-pipA-up-SE (*E. coli*)	χ7213 donor strain with pDM4-pipA-up-SE plasmid	This study
449/87-sifA-up-SE (*S*. Pullorum)	*S*. Pullorum 449/87 homo integrated with pDM4-sifA-up-SE plasmid by homologous recombination	
449/87-pipA-up-SE (*S*. Pullorum)	*S*. Pullorum 449/87 homo integrated with pDM4-pipA-up-SE plasmid by homologous recombination	
**Primers**		
DEL-SifA_F	taaaccctgaacgtgacgtttgagaaagcgtcgtctgattGTGTAGGCTGGAGCTGCTTC	Used for *sifA* inactivation by λ red recombination
DEL-SifA_R	attatgtagtcatttttactccagtttaagtgatattaatCATATGAATATCCTCCTTAG	

Plasmids, strains or primers	Description* or sequence (5'→3')	Source or comments
DEL-yeaG_F	ctgttctgtcagtgccggatagcgctaatgcttagccagtGTGTAGGCTGGAGCTGCTTC	Used for *yeaG* inactivation by λ red recombination
DEL-yeaG_R	ggctcttaacatgtgcgaaaaaacgaaaggatggcatatcCATATGAATATCCTCCTTAG	
DEL-SseE_F	cggttcttgctgaaatactccgggcattaacgaggtaaacGTGTAGGCTGGAGCTGCTTC	Used for *sseE* inactivation by λ red recombination
DEL-SseE_R	gttattttcacgtgataatgatttacgttaggaaggtcatCATATGAATATCCTCCTTAG	
DEL-OsmB_F	gagcttaattccgtgaatcgttaattcaggagagagtattGTGTAGGCTGGAGCTGCTTC	Used for *osmB* inactivation by λ red recombination
DEL-OsmB_R	aacgtattattttgagccgttcaatatttaaacgtctggaCATATGAATATCCTCCTTAG	
DEL-g5437_F	ttaaatacatcttttaatcaccacatcagggagatgtcttGTGTAGGCTGGAGCTGCTTC	Used for *group5437* inactivation by λ red recombination
DEL-g5437_R	ctgggcgggacgccgcccagtggtgactggtttaccgatgCATATGAATATCCTCCTTAG	
DEL-pagO_F	agctaaaaagctaaaataattttttttacggagaggatcttGTGTAGGCTGGAGCTGCTTC	Used for *pagO* inactivation by λ red recombination
DEL-pagO_R	cagtaaggcatcaggaattatttgcgttactataaatcgaCATATGAATATCCTCCTTAG	
DEL-g6178_F	tgccttaatcatatcttccgcgataacacagtttgtcaacGTGTAGGCTGGAGCTGCTTC	Used for *group6178* inactivation by λ red recombination
DEL-g6178_R	agtctctatattatgctttccccgctttacggagatgatgCATATGAATATCCTCCTTAG	
DEL-PipA_F	atttaaataattcataattgtagtcaggaaataagaagttGTGTAGGCTGGAGCTGCTTC	Used for *pipA* inactivation by λ red recombination
DEL-PipA_R	actacgtgggtttttagtttcttttcgtttcctgatgtgtCATATGAATATCCTCCTTAG	
DEL-tolQ_F	taagttcgcgacgacctcgtccacgcgttctggccatggcGTGTAGGCTGGAGCTGCTTC	Used for *tolQ* inactivation by λ red recombination
DEL-tolQ_R	cttgtgcgcttcctaagtctattgtcgcggagtttaagcaCATATGAATATCCTCCTTAG	
DEL-g4046_F	gaaacttgtcttttagcccaatattaaggcaggttctgaaGTGTAGGCTGGAGCTGCTTC	Used for *group4046* inactivation by λ red recombination
DEL-g4046_R	cttcgggaacccacaggaccagctatttttccgatagtgtCATATGAATATCCTCCTTAG	
DEL-SptP_F	gcttactttcagatagttctaaaagtaagctatgtttttaGTGTAGGCTGGAGCTGCTTC	Used for *sptP* inactivation by λ red recombination

Plasmids, strains or primers	Description* or sequence (5’→3’)	Source or comments
DEL-SptP_R	aacatactgcaggaatatgctaaagtatgaggagagaaaaCATATGAATATCCTCCTTAG	
DEL-glpD_F	tatgtggcgaaaagggcggtataaaccgccccggattttaGTGTAGGCTGGAGCTGCTTC	Used for *glpD* inactivation by λ red recombination
DEL-glpD_R	cgaacatttttgaactttaacgaaagtgcaagagggcagcCATATGAATATCCTCCTTAG	
DEL-g5201_F	ctttgcttccctctgattaccactagcgcaggatggcgcaGTGTAGGCTGGAGCTGCTTC	Used for *group5201* inactivation by λ red recombination
DEL-g5201_R	gtatagacaggatcgtttattctgaatagactaacgctttCATATGAATATCCTCCTTAG	
SNP-pipA-F	GAGCGGATAACAATTTGTGGAATCCCGGGAcggaggatagttcatcgtag	Construction of the pDM4-pipA-up-SE suicide plasmid used for A-$T^{449/87}$ → C-$G^{P125109}$ substitution in the upstream of *pipA* gene of 449/87 by allelic exchange
SNP-pipA-R	AGCGGAGTGTATATCAAGCTTATCGATACCagcgaaatcattaacaggag	
SNP-sifA-F	GAGCGGATAACAATTTGTGGAATCCCGGGAgcaacgctaacaaatccaca	Construction of the pDM4-sifA-up-SE suicide plasmid used for A-$T^{449/87}$ → C-$G^{P125109}$ substitution in the upstream of *sifA* gene of 449/87 by allelic exchange
SNP-sifA-R	AGCGGAGTGTATATCAAGCTTATCGATACCgcaggctaactctttcaactca	
k1	CAGTCATAGCCGAATAGCCT	(Datsenko & Wanner, 2000)

*Relevant antibiotic resistance are indicated by^{R}: Gem, gentamicin; Kam, kanamiycin; Amp, Ampicillin; Cm, chloramphenicol

References

Datsenko KA & Wanner BL (2000) One-step inactivation of chromosomal genes in Escherichia coli K-12 using PCR products. *Proc Natl Acad Sci U S A* **97**: 6640-6645.

Doublet B, Douard G, Targant H, Meunier D, Madec JY & Cloeckaert A (2008) Antibiotic marker modifications of lambda Red and FLP helper plasmids, pKD46 and pCP20, for inactivation of chromosomal genes using PCR products in multidrug-resistant strains. *J Microbiol Methods* **75**: 359-361.

Kang HY, Srinivasan J & Curtiss R, 3rd (2002) Immune responses to recombinant pneumococcal PspA antigen delivered by live attenuated Salmonella enterica serovar typhimurium vaccine. *Infect Immun* **70**: 1739-1749.

Milton DL, Norqvist A & Wolf-Watz H (1992) Cloning of a metalloprotease gene involved in the virulence mechanism of Vibrio anguillarum. *Journal of bacteriology* **174**: 7235-7244.

Yin C, Xu L, Li Y, Liu Z, Gu D, Li Q & Jiao X (2018) Construction of pSPI12-cured Salmonella enterica serovar Pullorum and identification of IpaJ as an immune response modulator. *Avian pathology : journal of the WVPA* **47**: 410-417.

Table S2. List of primers for genes expression analysis in avian HD11 cell line and *Salmonella* by RT-qPCR method.

Primer name	Sequence (5' to 3')	Target RNA	Reference
**HD11 cell line**			
GAPDH-F	GTCAGCAATGCATCGTGCA	GAPDH (internal control)	(Berndt *et al.*, 2007)
GAPDH-R	GGCATGGACAGTGGTCATAAGA		
IL-1β-F	GAAGTGCTTCGTGCTGGAGT	IL-1β	(Elsheimer-Matulova *et al.*, 2015)
IL-1β-R	ACTGGCATCTGCCCAGTTC		
IFN-γ-F	GCCGCACATCAAACACATATCT	IFN-γ	(Elsheimer-Matulova *et al.*, 2015)
IFN-γ-R	TGAGACTGGCTCCTTTTCCTT		
iNOS-F	TTGGAAACCAAAGTGTGTAATATCTTG	iNOS	(Tang *et al.*, 2018)
iNOS-R	CCCTGGCCATGCGTACAT		
IL-10-F	CGGGAGCTGAGGGTGAA	IL-10	(Hong *et al.*, 2006)
IL-10-R	GTGAAGAAGCGGTGACAGC		
IL-6-F	GCGAGAACAGCATGGAGATG	IL-6	(Jiang *et al.*, 2011)
IL-6-R	GTAGGTCTGAAAGGCGAACAG		
TGF-β4-F	AGGATCTGCAGTGGAAGTGGAT	TGF-β4	(Tang *et al.*, 2018)
TGF-β4-R	CCCCGGGTTGTGTGTTGGT		
Salmonella			
Gmk-ref-F	ACACTCAGGTTTCCGTTTCAC	*gmK* (internal control)	(Botteldoorn *et al.*, 2006)
Gmk-ref-R	GCACTTGCTCAATGGTTTCG		

**Primer name**	**Sequence (5’ to 3’)**	**Target RNA**	**Reference**
PipA-F	ACGGCTACGCCTTGCTC	*pipA*	This study
PipA-R	CTGTATCAGGGTCTATTCCAC		
SifA-F	TCAGGCGTTCCTCGTAA	*sifA*	This study
SifA-R	AGCCGCTTTGTTGTTCT		
SptP-F	ATGAGCGGACCGACACT	*sptP*	This study
SptP-R	TACGAACCGGCTAATGC		
YgbK-F	GATACCTCATTTACCGTGATTTCTCC	*ygbK*	This study
YgbK-R	GTCATCGGATTGATTGGGTGG		
AroL-F	GTGTTGGCGTAGTGATTTATCTGTG	*aroL*	This study
AroL-R	CTTTCGTCGCGTCAATAATGTAAT		
Nfo-F	CGACGGCGTGGAAGAT	*nfo*	This study
Nfo-R	GCAAATACTGAAATCCGACAAT		
SseE-F	GTCGGCATCAGGTGTT	*sseE*	This study
SseE-R	TGCTGTGGTCGAGGTT		
SopB-F	CTGGGTTGGCGAATG	*sopB*	This study
SopB-R	GCGTTGTGCGAGTTTAT		

References

Berndt A, Wilhelm A, Jugert C, Pieper J, Sachse K & Methner U (2007) Chicken cecum immune response to Salmonella enterica serovars of different levels of invasiveness. *Infect Immun* **75**: 5993-6007.

Botteldoorn N, Van Coillie E, Grijspeerdt K, Werbrouck H, Haesebrouck F, Donne E, D'Haese E, Heyndrickx M, Pasmans F & Herman L (2006) Real-time reverse transcription PCR for the quantification of the mntH expression of Salmonella enterica as a function of growth phase and phagosome-like conditions. *J Microbiol Methods* **66**: 125-135.

Elsheimer-Matulova M, Varmuzova K, Kyrova K, Havlickova H, Sisak F, Rahman M & Rychlik I (2015) phoP, SPI1, SPI2 and aroA mutants of Salmonella Enteritidis induce a different immune response in chickens. *Vet Res* **46**: 96.

Hong YH, Lillehoj HS, Lillehoj EP & Lee SH (2006) Changes in immune-related gene expression and intestinal lymphocyte subpopulations following Eimeria maxima infection of chickens. *Vet Immunol Immunopathol* **114**: 259-272.

Jiang H, Yang H & Kapczynski DR (2011) Chicken interferon alpha pretreatment reduces virus replication of pandemic H1N1 and H5N9 avian influenza viruses in lung cell cultures from different avian species. *Virology journal* **8**: 447.

Tang Y, Foster N, Jones MA & Barrow PA (2018) Model of Persistent Salmonella Infection: Salmonella enterica Serovar Pullorum Modulates the Immune Response of the Chicken from a Th17-Type Response towards a Th2-Type Response. *Infect Immun* **86**: e00307-00318.

Table S3. List of putative T3SE genes identified by the custom-build bioinformatics workflow (by route A).

TAG_name	preferred_name (Eggnog_v2)	Annotation (Eggnog_v2)
aroL	aroL	Catalyzes the specific phosphorylation of the 3-hydroxyl group of shikimic acid using ATP as a cosubstrate
nfo	nfo	Endonuclease IV plays a role in DNA repair. It cleaves phosphodiester bonds at apurinic or apyrimidinic sites (AP sites) to produce new 5'-ends that are base-free deoxyribose 5-phosphate residues. It preferentially attacks modified AP sites created by bleomycin and neocarzinostatin
pipA	pipA	PipA protein
sifA	sifA	Sif protein
sopB	sopB	Enterobacterial virulence protein
sptP	sptP	Similar to Yersinia virulence determinant
sseE	sseE	Type III secretion system regulator
ygbK	ygbK	Putative sugar-binding N-terminal domain

Table S4. List of genes with immune epitopes identified by the custom-build bioinformatics workflow (by route B).

Epitope_ID	QUERY_LOCUS_TAG	Epitope sequence	Antigen Name	Antigen Accession	Organism Name
55468	alaS	RQQAQVEQVLKTEEEQFARTLERGLALLDEELAKLS	Alanyl-tRNA synthetase (Alanine--tRNA ligase) (AlaRS)	P00957.2	Escherichia coli K-12
737500	asnA	RGEMPQTIGGGIGQSRL	aspartate--ammonia ligase [Salmonella enterica]	WP_072643073.1	Salmonella enterica
124848	ccmF	VVNDFTVSY	cytochrome c-type biogenesis protein	ABG18536.1	Yersinia pestis Nepal516
14759	clpB	EVLARWTGIPVS	ATP-dependent protease binding subunit	AAA24422.1	Escherichia coli
126022	clpB	FMFDSDEAM	ClpB protein	ABG70584.1	Escherichia coli 536
737879	cyoA	VAKAKQSPNTMNDMAAFEKVAMPSEYN	Cytochrome O ubiquinol oxidase subunit II [Salmonella enterica subsp. enterica serovar Typhimurium]	APQ79374.1	Salmonella enterica
229438	dnaT_1	QFYEEPEAAPVAI	primosomal protein I	AAL23362.1	Salmonella enterica subsp. enterica serovar Typhimurium str. LT2
69954	fdoG	VMGGNAAEA	Formate dehydrogenase-N, nitrate-inducible, alpha subunit	Q8XAS2	Escherichia coli O157:H7
124915	folC	YSSPHLLRY	FolC bifunctional protein	ABG18507.1	Yersinia pestis Nepal516
70469	glpD	VPRVHNQPQ	aerobic glycerol-3-phosphate dehydrogenase (partial)	CAL22518.1	Yersinia pestis CO92
124099	group_4264	FERGVINVF	unknown	AAG55370.1	Escherichia coli O157:H7 str. EDL933
124091	group_5201	FAHDDRYLY	putative exported protein	CAD06877.1	Salmonella enterica subsp. enterica serovar Typhi str. CT18
124870	group_5201	WVAGVQLLY	putative exported protein	CAD06877.1	Salmonella enterica subsp. enterica serovar Typhi
124375	group_5201	KQFCLSILL	putative exported protein	CAD06877.1	Salmonella enterica subsp. enterica serovar Typhi str. CT18
124902	group_5201	YQVKYVSPV	putative exported protein	CAD06877.1	Salmonella enterica subsp. enterica serovar Typhi str. CT18
124293	group_5201	HVAAFSSLI	putative exported protein	CAD06877.1	Salmonella enterica subsp. enterica serovar Typhi str. CT18
124088	group_5201	FAADKDSLY	putative exported protein	CAD06877.1	Salmonella enterica subsp. enterica serovar Typhi str. CT18
124735	group_5201	SPVDRVLTI	putative exported protein	CAD06877.1	Salmonella enterica subsp. enterica serovar Typhi str. CT18
36655	group_5260	LIPDGDGEV	putative beta-glucosidase	BAB92992.1	Serratia marcescens
126477	group_5437	KRSTPFYTK	conserved hypothetical protein	CAD05491.1	Salmonella enterica subsp. enterica serovar Typhi

Epitope_ID	QUERY_LOCUS_TAG	Epitope sequence	Antigen Name	Antigen Accession	Organism Name
229478	group_6161	SAYLGPVAVLVDT	PTS family galactitol-specific enzyme IIC	AAL22132.1	Salmonella enterica subsp. enterica serovar Typhimurium str. LT2
92754	group_6331	FTGEYLLRL	putative voltage-gated potassium channel	AAL20659.1	Salmonella enterica subsp. enterica serovar Typhimurium str. LT2
93713	gsk	RIYSHIAPY	inosine-guanosine kinase	ABG17326.1	Yersinia pestis Nepal516
229217	iadA	GAYHVPSPTITGS	isoaspartyl dipeptidase	AAL23330.1	Salmonella enterica subsp. enterica serovar Typhimurium str. LT2
126155	katG	GMRVLGTNY	catalase/peroxidase	EAX57207.1	Vibrio cholerae 2740-80
52493	moeA	QTDDGVRFT	molybdopterin biosynthesis protein	AAL19782.1	Salmonella enterica subsp. enterica serovar Typhimurium str. LT2
30735	mopA	KFGNDARIKMLRGVNILA	60 kDa chaperonin	P48219.1	Yersinia enterocolitica
21080	mopA	GLQDELDVVEGMQFDRG	60 kDa chaperonin	P0A6F5.2	Escherichia coli
43581	mopA	NEDQNVGIKVALRAMEA	60 kDa chaperonin	P0A6F5.2	Escherichia coli
54170	mopA	RIKMLRGVNILADAVKVT	60 kDa chaperonin	P48219.1	Yersinia enterocolitica
69048	mopA	VINKDTTIIIDGVG	60 kDa chaperonin	P48219.1	Yersinia enterocolitica
13043	mopA	ELESPFILLADK	60 kDa chaperonin	P48219.1	Yersinia enterocolitica
9104	mopA	DLGQAKRVVIN	60 kDa chaperonin	P48219.1	Yersinia enterocolitica
21327	mopA	GMQFDRGYL	60 kDa chaperonin	P0A1D3.2	Salmonella enterica subsp. enterica serovar Typhimurium str. LT2
56441	mopA	RVVINKDTTIII	60 kDa chaperonin	P48219.1	Yersinia enterocolitica
150991	mopA	GRNVVLDKS	60 kDa chaperonin	P48219.1	Yersinia enterocolitica
3152	mopA	AMLQDIATL	60 kDa chaperonin	P0A1D4.2	Salmonella typhi Ty21a
5258	mopA	AVAKAGKPL	60 kDa chaperonin	P0A1D4.2	Salmonella typhi Ty21a
68192	mopA	VEGEALATL	60 kDa chaperonin	P0A1D4.2	Salmonella typhi Ty21a
33245	mopA	KRVVINKDT	60 kDa chaperonin	P48219.1	Yersinia enterocolitica
225393	mopA	LEDKFENMGAQMVKE	60 kDa chaperonin GROEL	AAO91213.1	Coxiella burnetii
225265	mopA	EDKFENMGAQMVKEV	60 kDa chaperonin GROEL	AAO91213.1	Coxiella burnetii
736780	mopA	GPKGRNVVLDKSFGAPTITKDGVS	chaperonin GroEL [Salmonella enterica]	WP_070803993.1	Salmonella enterica
738001	mopA	VVLDKSFGAPTITKDGVS	chaperonin GroEL [Salmonella enterica]	WP_070803993.1	Salmonella enterica
737597	mopA	SFGAPTITKDG	chaperonin GroEL [Salmonella enterica]	WP_070803993.1	Salmonella enterica
32361	mopA	KMLRGVNVL	chaperonin GroEL [Salmonella enterica]	WP_070803993.1	Salmonella enterica

Epitope_ID	QUERY_LOCUS_TAG	Epitope sequence	Antigen Name	Antigen Accession	Organism Name
496689	mopA	LATLVVNTMRGIVKVAAVKAPGFGDRRKAM	Cpn60 chaperonin GroEL, large subunit of GroESL	AAC77103.1	Escherichia coli
231292	mopA	PCSDSKAIAQVGTISANSDE	GroEL	AAS75782.1	Escherichia coli
119789	mopA	DARVKMLRGVNVL	GroEL	AAS75782.1	Escherichia coli
496685	mopA	LATLVVNNMRGIVKVCAVKAPGFGDRRKAM	molecular chaperone GroEL	YP_126086.1	Legionella pneumophila
109605	nfo	MQDGRFDGI	Probable endonuclease 4	Q8ZNK6.1	Salmonella enterica subsp. enterica serovar Typhimurium
226020	nmpC	FAGLKYADVGSFDYGRNYGV	outer membrane porin 1a (Ia;b;F)	NP_415449.1	Escherichia coli
55063	nmpC	RNTDFFGL	outer membrane porin C precursor - Salmonella typhimurium	A59139	Salmonella enterica subsp. enterica serovar Typhimurium
31787	nmpC	KLDLYGKVH	Outer membrane porin protein ompD precursor	P37592.2	Salmonella enterica subsp. enterica serovar Typhimurium
15183	nmpC	FAGLKFADY	Outer membrane porin protein ompD precursor	P37592.2	Salmonella enterica subsp. enterica serovar Typhimurium
27681	nmpC	INLLDDSDF	Outer membrane porin protein ompD precursor	P37592.2	Salmonella enterica subsp. enterica serovar Typhimurium
25483	nmpC	IAYLKSKGK	Outer membrane porin protein ompD precursor	P37592.2	Salmonella enterica subsp. enterica serovar Typhimurium
1309301	nmpC	VGATYYFNKNMSTYVDYKIN	porin OmpC [Escherichia coli]	WP_137568092.1	Escherichia coli
109825	repC	RIYRKGNPL	CopB/RepC	BAB20532.1	Salmonella enterica subsp. enterica serovar Choleraesuis
736286	tolQ	AFIALGAVKQATLQMVAPGIAEAL	protein TolQ [Salmonella enterica]	WP_072224641.1	Salmonella enterica
92587	torD	EPADHLAIM	TorD protein	BAC95068.1	Vibrio vulnificus YJ016
127211	tus	TKEAEHAPL	DNA replication terminus site-binding protein; DNA sequence-specific contrahelicase	CAD01897.1	Salmonella enterica subsp. enterica serovar Typhi
229423	yafJ	PSYHSPIAKLVQN	putative glutamine amidotransferase	AAL19268.1	Salmonella enterica subsp. enterica serovar Typhimurium str. LT2
92555	ycdW	EIIFYHPTF	putative oxidoreductase	AAL20065.1	Salmonella enterica subsp. enterica serovar Typhimurium str. LT2
737021	yeaG	KEPENSSIYSKMRVYDG	PrkA family serine protein kinase [Salmonella enterica]	WP_070810756.1	Salmonella enterica
126968	yeiR	RQGKPDWLL	Unknown	AAO10720.1	Vibrio vulnificus CMCP6
126877	yicI	QKMHNHYAY	alpha-xylosidase	ABV08072.1	Escherichia coli HS

Table S5. List of putative genes of importance for persistent infection identified by the custom-build bioinformatics workflow (by route C).

**query**	**Description**	**Preferred_name**	**PFAMs**
sinH	Pfam:Invasin_beta	-	IAT_beta
safC_1	PapC C-terminal domain	safC	PapC_C,PapC_N,Usher
abc	Part of the ABC transporter complex MetNIQ involved in methionine import. Responsible for energy coupling to the transport system	metN	ABC_tran,NIL
yafE	Putative S-adenosyl-L-methionine-dependent methyltransferase	yafE	Methyltransf_11,Methyltransf_25
sinR	LysR substrate binding domain	-	HTH_1,LysR_substrate
stiH	Fimbrial protein	-	Fimbrial
stfF_2	Fimbrial protein	-	Fimbrial
gltL	ABC transporter	gltL	ABC_tran
group_5107	Sugar (and other) transporter	yebQ	MFS_1
stfG_2	Fimbrial protein	-	Fimbrial
group_1317	Catalyzes the transfer of sulfuryl groups between phenolic compounds	-	Arylsulfotran_N,Arylsulfotrans
yafC_1	LysR substrate binding domain	yafC	HTH_1,LysR_substrate
yieO_1	Transmembrane secretion effector	hsrA	MFS_1,MFS_3,Sugar_tr
group_5759	LysR substrate binding domain	-	HTH_1,LysR_substrate
ugpC	Part of the ABC transporter complex UgpABCE involved in sn-glycerol-3-phosphate import. Responsible for energy coupling to the transport system	ugpC	ABC_tran,TOBE_2
group_6312	PTS system, Lactose/Cellobiose specific IIB subunit	sgcB	PTS_IIB
group_5206	Major facilitator superfamily	-	MFS_1
ygiY	His Kinase A (phospho-acceptor) domain	qseC	2CSK_N,HATPase_c,HisKA

query	Description	Preferred_name	PFAMs
ecnR	Bacterial regulatory proteins, luxR family	ecnR	GerE
znuC	Part of the ABC transporter complex ZnuABC involved in zinc import. Responsible for energy coupling to the transport system	znuC	ABC_tran
torD	Involved in the biogenesis of TorA. Acts on TorA before the insertion of the molybdenum cofactor and, as a result, probably favors a conformation of the apoenzyme that is competent for acquiring the cofactor	torD	Nitrate_red_del
ybjG	PAP2 superfamily	ybjG	PAP2
aroL	Catalyzes the specific phosphorylation of the 3-hydroxyl group of shikimic acid using ATP as a cosubstrate	aroL	SKI
ybeF	LysR substrate binding domain	ybeF	HTH_1,LysR_substrate
setB	MFS_1 like family	setB	MFS_1
yfhB	haloacid dehalogenase-like hydrolase	yfhB	HAD
group_4738	Helix-turn-helix domain	envY	HTH_18,HTH_AraC
group_4620	Major Facilitator Superfamily	ygaY	MFS_1,Sugar_tr
stbE	Pili assembly chaperone PapD, C-terminal domain	fimB	PapD_C,PapD_N
group_4046	Opacity family porin protein	tia	OMP_b-brl
stiC_2	PapC C-terminal domain	-	PapC_C,PapC_N,Usher
cspD	'Cold-shock' DNA-binding domain	cspD	CSD
group_1391	CheW-like domain	cheV	CheW,Response_reg
entB	Phosphopantetheine attachment site	entB	Isochorismatase,PP-binding
manC	Mannose-6-phosphate isomerase	cpsB	MannoseP_isomer,NTP_transferase
tdh_1	Glucose dehydrogenase C-terminus	ydjL	ADH_N,ADH_zinc_N
group_6424	Uncharacterised MFS-type transporter YbfB	ydhP	MFS_1,Sugar_tr
group_1570	MFS_1 like family	mdtG	MFS_1,Sugar_tr

query	Description	Preferred_name	PFAMs
ybiF	EamA-like transporter family	rhtA	EamA
glpD	C-terminal domain of alpha-glycerophosphate oxidase	glpD	DAO,DAO_C
stfD_1	Pili assembly chaperone PapD, C-terminal domain	mrfD	PapD_C,PapD_N
ilvA	Catalyzes the anaerobic formation of alpha-ketobutyrate and ammonia from threonine in a two-step reaction. The first step involved a dehydration of threonine and a production of enamine intermediates (aminocrotonate), which tautomerizes to its imine form (iminobutyrate). Both intermediates are unstable and short- lived. The second step is the nonenzymatic hydrolysis of the enamine imine intermediates to form 2-ketobutyrate and free ammonia. In the low water environment of the cell, the second step is accelerated by RidA	ilvA	PALP,Thr_dehydrat_C
sifA	Sif protein	sifA	Sif
nfo	Endonuclease IV plays a role in DNA repair. It cleaves phosphodiester bonds at apurinic or apyrimidinic sites (AP sites) to produce new 5'-ends that are base-free deoxyribose 5-phosphate residues. It preferentially attacks modified AP sites created by bleomycin and neocarzinostatin	nfo	AP_endonuc_2
group_5481	LysR substrate binding domain	cynR	HTH_1,LysR_substrate
fixC	part of an electron transfer system required for anaerobic carnitine reduction	fixC	DAO,FAD_binding_3,NAD_binding_8
stbC_2	PapC C-terminal domain	stbC	PapC_C,PapC_N,Usher
yaiC	MASE2 domain	adrA	GGDEF,MASE2
group_6178	SH3 domain (SH3b1 type)	-	NLPC_P60,N_NLPC_P60,SH3_6,SH3_7
lpfA	Fimbrial protein	lpfA	Fimbrial
group_4932	HlyD membrane-fusion protein of T1SS	-	Biotin_lipoyl_2,HlyD,HlyD_3
group_1282	Bacterial regulatory helix-turn-helix protein, lysR family	-	HTH_1,LysR_substrate
yiaK	Catalyzes the reduction of 2,3-diketo-L-gulonate in the presence of NADH, to form 3-keto-L-gulonate	dlgD	Ldh_2
group_5127	Periplasmic sensor domain	yedQ	CHASE7,GGDEF

query	Description	Preferred_name	PFAMs
nagZ	Plays a role in peptidoglycan recycling by cleaving the terminal beta-1,4-linked N-acetylglucosamine (GlcNAc) from peptide-linked peptidoglycan fragments, giving rise to free GlcNAc, anhydro-N-acetylmuramic acid and anhydro-N-acetylmuramic acid-linked peptides	nagZ	Glyco_hydro_3
pagO	EamA-like transporter family	pagO	EamA
finP	Transglycosylase SLT domain	-	SLT
cyoA	Cytochrome C oxidase subunit II, periplasmic domain	cyoA	COX2,COX_ARM
group_1421	Sugar (and other) transporter	-	Sugar_tr
hemY	HemY protein N-terminus	hemY	HemY_N,TPR_2
group_3804	Domain of unknown function (DUF4156)	-	DUF4156
napG	4Fe-4S binding domain	napG	Fer4,Fer4_4,Fer4_7
group_6316	Protein of unknown function (DUF3829)	-	DUF3829
ynbE	YnbE-like lipoprotein	ynbE	Lipoprotein_19
ydjE_2	Sugar (and other) transporter	ydjE	Sugar_tr
yiaM	Tripartite ATP-independent periplasmic transporters, DctQ component	yiaM	DctQ
cueO	Multicopper oxidase	cueO	Cu-oxidase,Cu-oxidase_2,Cu-oxidase_3,TAT_signal
aegA	Dihydroprymidine dehydrogenase domain II, 4Fe-4S cluster	gltD	Fer4_20,Pyr_redox_2
group_5201	-	-	-
frdB	2Fe-2S iron-sulfur cluster binding domain	frdB	Fer2_3,Fer4_8
osmB	Glycine zipper 2TM domain	osmB	Rick_17kDa_Anti
nlpE	NlpE C-terminal OB domain	cutF	META,NlpE,NlpE_C
group_5866	NiFe/NiFeSe hydrogenase small subunit C-terminal	hyaA	NiFe_hyd_SSU_C,Oxidored_q6

query	Description	Preferred_name	PFAMs
sscB	Tetratricopeptide repeat	sscB	-
nlpD	Lysin motif	nlpD	LysM,Peptidase_M23
dmsA_1	-	-	-
